# Assembly of Bak homodimers into higher order homooligomers in the mitochondrial apoptotic pore

**DOI:** 10.1038/srep30763

**Published:** 2016-08-04

**Authors:** Tirtha Mandal, Seungjin Shin, Sreevidya Aluvila, Hui-Chen Chen, Carter Grieve, Jun-Yong Choe, Emily H. Cheng, Eric J. Hustedt, Kyoung Joon Oh

**Affiliations:** 1 Department of Biochemistry and Molecular Biology, Rosalind Franklin University of Medicine and Science, North Chicago, Illinois 60064, USA; 2 Human Oncology and Pathogenesis Program and Department of Pathology, Memorial Sloan Kettering Cancer Center, New York, New York 10065, USA; 3 Molecular Physiology and Biophysics, Vanderbilt University School of Medicine, Nashville, Tennessee 37232, USA

## Abstract

In mitochondrial apoptosis, Bak is activated by death signals to form pores of unknown structure on the mitochondrial outer membrane via homooligomerization. Cytochrome *c* and other apoptotic factors are released from the intermembrane space through these pores, initiating downstream apoptosis events. Using chemical crosslinking and double electron electron resonance (DEER)-derived distance measurements between specific structural elements in Bak, here we clarify how the Bak pore is assembled. We propose that previously described BH3-in-groove homodimers (BGH) are juxtaposed via the ‘α3/α5’ interface, in which the C-termini of helices α3 and α5 are in close proximity between two neighboring Bak homodimers. This interface is observed concomitantly with the well-known ‘α6:α6’ interface. We also mapped the contacts between Bak homodimers and the lipid bilayer based on EPR spectroscopy topology studies. Our results suggest a model for the lipidic Bak pore, whereby the mitochondrial targeting C-terminal helix does not change topology to accommodate the lining of the pore lumen by BGH.

B cell lymphoma-2 (Bcl-2) family proteins are central regulators in the mitochondrial apoptosis pathway^1^,^2^,^3^,^4^. Among them, the multi-domain proapoptotic Bcl-2 proteins such as Bax (Bcl-2-associated X protein) and Bak (Bcl-2 antagonist/killer) are the gateway to mitochondrial dysfunction and cell death^5^ (see Supplementary Information Figure S1a). Bax remains in the cytoplasm before it is activated by cell death signals and translocates to the mitochondrial outer membrane^6^. Bak is held in check by voltage-dependent anion channel 2, Mcl-1, or Bcl-x_L_ in the mitochondrial outer membrane before its activation by death signals^7^,^8^. Upon activation^9^,^10^,^11^,^12^,^13^, Bax and Bak oligomerize and permeabilize the mitochondrial outer membrane by forming large pores^14^,^15^,^16^,^17^,^18^,^19^,^20^,^21^. Through these pores, which have the shapes of rings in super-resolution microscopy^18^,^19^, apoptotic factors including cytochrome *c* are released into the cell cytoplasm from the mitochondrial intermembrane space^22^.

Various biochemical and biophysical studies have shown that Bax and Bak form homodimers first and they further oligomerize to form pores^9^,^15^,^23^,^24^,^25^,^26^,^27^,^28^. The core of the human Bax or Bak homodimer, known as “BH3-in-groove homodimer (BGH),” is formed by symmetric association of two identical polypeptides consisting of helices α2-α5^25^,^29^. In BGH, two identical extended α2-α3 helices are arranged in an anti-parallel orientation forming an upper hydrophilic surface while two helical hairpins made of α4-α5, also arranged in anti-parallel orientation, form a lower hydrophobic face that is in contact with the membrane in human Bax and Bak^25^,^29^,^30^. Additionally, α6 and α9 helices form the interfaces between the BGHs, known as ‘α6:α6 interface’ and ‘α9:α9 interface,’ respectively^23^,^31^. It was also hypothesized that α6 helices line the oligomeric Bak pore^30^. Contrary to this, a ‘clamp model’ was proposed for Bax in which the BGHs line the lipidic pore while the α6 helices ‘clamp’ the flat region of the membrane at the periphery of the pore^32^. Thus, how the Bax and Bak homodimers are organized in oligomeric pore remains controversial and unclear.

Previously, we found that the mouse BGH structure exists in oligomeric pores formed in liposomes^27^,^33^. We also reported evidence that the BGHs are assembled via a novel oligomerization interface that involve the C-termini of helices α3 and α5, which were termed ‘α3:α3’, α5:α5’ oligomerization interface’ (‘α3/α5 interface,’ hereafter)^27^. However, these were demonstrated in the artificial liposomal systems and evidences from the apoptotic mitochondria were lacking. Furthermore, due to the lack of the BGH structure of mouse Bak, we had to rely on a homology model to interpret our data. In this current study, the X-ray crystal structure of BGH containing helices α2-α5 from mouse Bak is presented and the existence of the ‘α3/α5 interface’ in oligomeric Bak is demonstrated by chemical cross-linking approach using the mitochondria isolated from the mouse embryonic fibroblast (MEF) cells that express various Bak cysteine substitution mutants. The membrane immersion depths of selected amino acid residues in the hydrophobic surface of the BGH and in α6 helix are also presented along with the double electron electron resonance (DEER) data consistent with the ‘α3/α5 interface’. These results, in combination with the previously known interfaces mentioned above, provide critical insights into the structure of apoptotic Bak pores.

## Results

### Mouse Bak helices α2-α5 also form BH3-in-groove homodimer (BGH)

**Figure 1:**
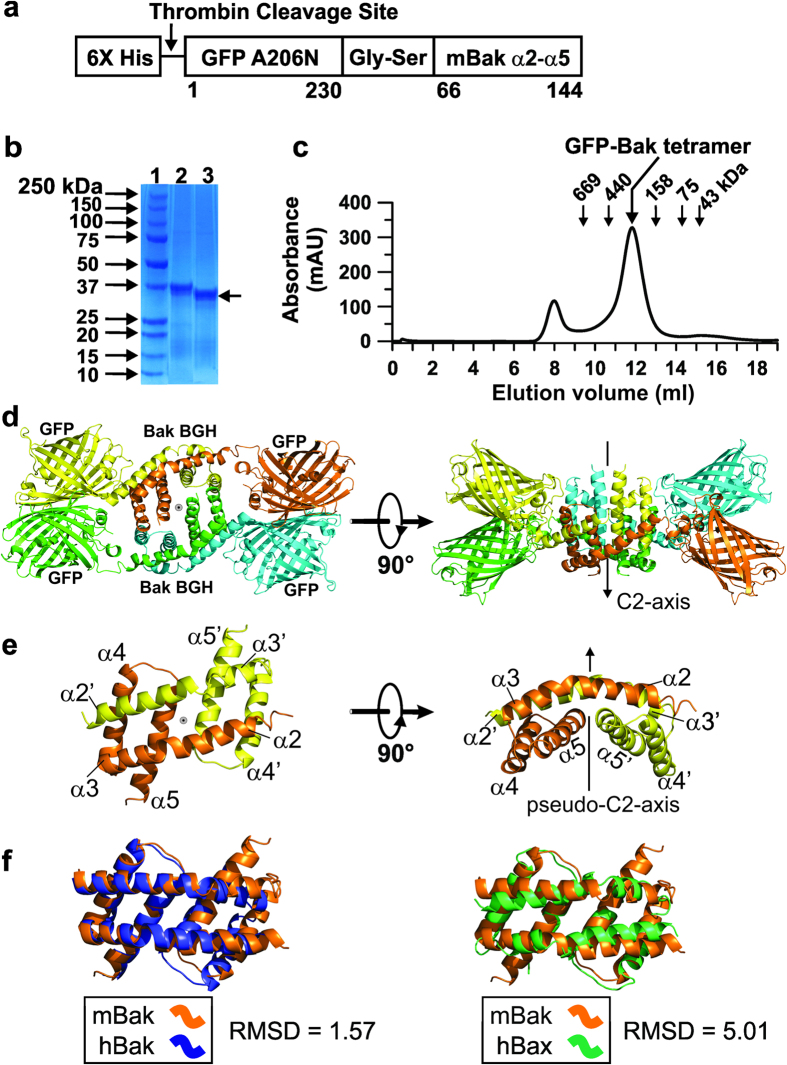
X-ray crystal structure of Bak BH3-in-groove homodimer (BGH). (**a**) Schematic representation of N-terminally hexahistidine tagged green fluorescent protein (GFP, residues 1–230) fused to the helices α2-α5 of mouse Bak (residues 66–144) (designated as His-GFP-Bak). The A206N mutation enables GFP to dimerize. (**b**) SDS-polyacrylamide gel electrophoresis of His-GFP-Bak before (lane 2) and after (lane 3, arrow) thrombin cleavage of His-tag under a reducing condition. (**c**) The peak corresponding to the GFP-Bak tetramer (~228 kDa) is shown in a gel filtration chromatogram (run at 0.5 ml/min using a Superdex 200 column (GE healthcare)) along with the positions of the indicated gel filtration standards. (**d**) A ribbon diagram of the GFP-Bak tetramer structure at 2.8 Å (PDB ID: 5KTG) in two orthogonal views. The backbones of GFP-Bak monomers are color-coded (orange, yellow, green and blue for A, B, C and D chains, respectively). (**e**) The ribbon diagram of the BGH structure. The BGH (A, B-chain) in (**d**) is shown in two orthogonal views with the two polypeptides color-coded the same as in (**d**). (**f**) BGH (A,B-chain) was aligned with the reported BGHs of human BAX (PDB ID: 4BDU)^25^ and the human BAK (PDB ID: 4U2V)^29^, respectively, using Pymol^59^. The root-mean-square deviation (RMSD) values for the color-coded polypeptide backbone chains were calculated using Pymol^59^.

An atomic resolution structure of the mouse BGH was needed to guide the site-directed spin labeling work presented here and for structural modeling of the oligomeric Bak pore. We thus first solved the X-ray crystal structure of BGH as described by others^29^,^34^. A fusion protein in which a hexahistidine-tagged dimerizable green fluorescent protein is fused to mouse Bak helices α2-α5 (designated as His-GFP-Bak) was expressed (Fig. 1a). The fusion protein was purified and the His-tag was removed by thrombin digestion (Fig. 1b, lane 3). The resulting protein, designated as GFP-Bak, was crystallized as described in the Methods. GFP-Bak existed as a tetramer with an apparent molecular weight (MW) of 228 kDa estimated by gel filtration chromatography (Fig. 1c), close to 210 (±20) kDa estimated by the quasi-elastic light scattering (QELS). The large deviation of the MW from the theoretical value of 141 kDa was due to the elongated shape of the tetramer (Fig. 1d).

GFP-Bak tetramers crystallized, solely mediated by the contacts between GFP molecules (Supplementary Information Figure S1b). The crystal structure of the GFP-Bak tetramer was refined to 2.8 Å resolution (Table 1 and Fig. 1d; PDB ID: 5KTG). In this structure, two GFP molecules were bridged by the mouse BGH, which in turn formed a tetramer around a two-fold symmetry axis (C2-axis) (Fig. 1d). The overall organization of the GFP-Bak tetramer was different from any of the GFP-BGH structures known^29^,^34^. Despite this, the folding of the mouse BGH itself was similar to that of the human Bak or Bax^29^,^34^ (Fig. 1e,f). The BGH unit was formed by two anti-parallel α2-α3 extended helices in the upper layer and the two α4-α5 helical hairpins symmetrically arranged in the lower layer (Fig. 1e). The backbone atom root-mean-square deviations (RMSD) values calculated between the mouse BGH and the human Bax and Bak BGH were 1.57 Å and 5.01, respectively (Fig. 1f), indicating that the mouse Bak BGH is similar to that of human Bak. The larger RMSD for human Bax was due to the twisting of the upper helical layer of Bax BGH relative to the lower one (Fig. 1f, right panel).

### The Bak homodimers oligomerize via ‘α3/α5 interface’ as well as ‘α6:α6 interface’ in mitochondria

**Figure 2:**
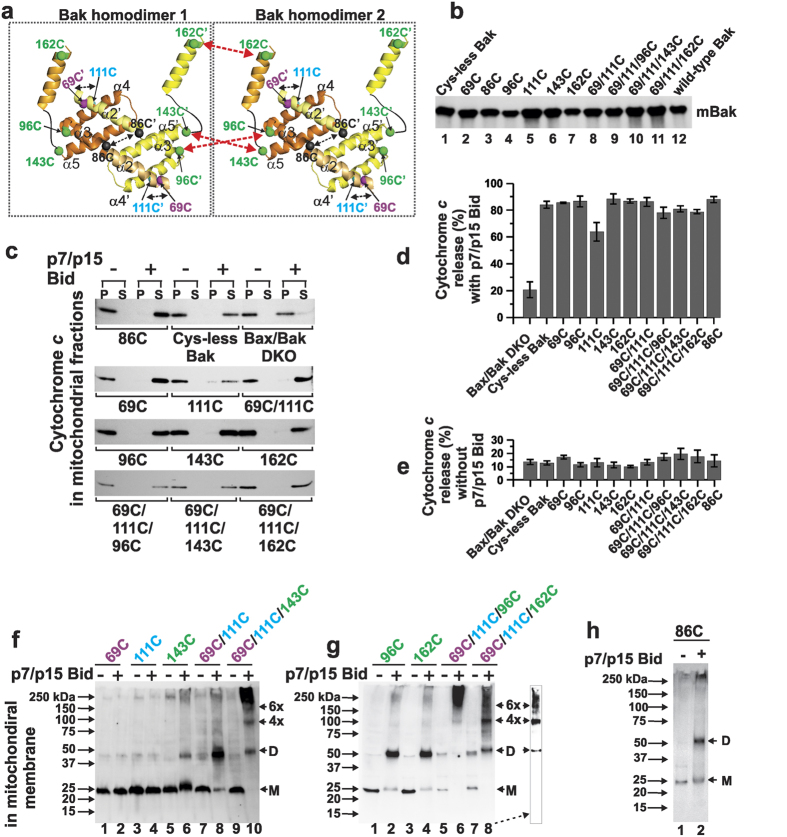
Bak homodimers oligomerize via ‘α3/α5 interface’ as well as ‘α6:α6 interface’ in mitochondrial apoptotic Bak pore. (**a**) Two Bak homodimers (each made of two Bak monomers in yellow and orange, with only α2-α6 helices shown) are brought to each other in the oligomeric Bak pore, forming the indicated inter-dimer disulfide bonds (red dotted arrows). The black dotted arrows represent the intra-dimer disulfide bonds. The helices and amino acid residues are labeled with and without prime (’) for the two identical polypeptide chains, respectively, in each BGH. (**b**) Expression of full length Bak cysteine substitution mutant proteins in the isolated mitochondria from MEF cells by PAGE/Western blot analysis (~30 μg mitochondrial proteins/lane). (**c**) Cytochrome *c* release activity of Bak cysteine mutant proteins in MEF mitochondria by PAGE/Western blotting. The cytochrome *c* remaining in the mitochondra (P) and that released into the medium (S) were determined after incubating the mitochondria (~30 μg protein) in the presence of 100 nM (or 0 nM) p7/p15 Bid for 30 min at 30 °C. (**d**,**e**) The percent of cytochrome *c* released from the mitochondria (average of two experiments, ± range). (**f**,**g**,**h**) Copper(II)(1,10-phenanthroline)_3_-catalyzed disulfide bond formation in various Bak cysteine mutants expressed in MEF mitochondria with or without activation by 100 nM p7/p15 Bid (also see Supplementary Information Figure S1c). The western blotting images were obtained after nonreducing SDS PAGE (~30 μg mitochondrial proteins/lane). The image of lane 8 with a reduced background is shown in the boxed inset to identify the bands of the higher order oligomeric Bak more clearly.

To determine how Bak homodimers oligomerize in the mitochondrial outer membrane, we mapped the proximity of amino acid residues in the Bak oligomeric pore using disulfide cross-linking^35^ (Fig. 2a). Stable expression of full length Bak mutants containing single, double and triple cysteine substitutions at strategic positions was performed in Bax^−/−^Bak^−/−^ mouse embryonic fibroblasts (MEFs) (see Methods). These Bak mutant proteins targeted to the mitochondria normally, as evidenced by the Western blot analysis (Fig. 2b). The parent cysteine-less Bak (lane 1, Fig. 2b) and the cysteine substitution mutants (lanes 2–11, Fig. 2b) were expressed in varying quantities relative to the wild-type Bak (lane 12, Fig. 2b) (from the lowest ~80% for 162C to the highest ~130% for 111C). These mutant proteins were active in apoptotic pore formation in the mitochondrial outer membrane, as evidenced by the efficient release of cytochrome *c* from the mitochondria (Fig. 2c–e). When the Bak proteins were activated by p7/p15 Bid, approximately 80–90 percent of the cytochrome *c* molecules were released from the mitochondria except for mutant 111C (Fig. 2c,d). In the absence of p7/p15 Bid, less than 20 percent of the cytochrome *c* was released in all the cases (Fig. 2c,e). These data indicated that the cysteine substitution Bak mutant proteins expressed in the MEF mitochondria were mostly intact in their structure and apoptotic function.

In the mouse BGH structure, the α-carbon atom (C_α_) of residue 69 on helix α2 in one α2-α5 polypeptide chain is in close proximity to the C_α_ of reside 111 on helix α4 in the other paired polypeptide (spheres in purple and cyan, respectively, Fig. 2a). The shortest distance between the β-carbon atoms of the cysteines introduced at these two locations is 4.6 Å in the BGHs of the GFP-Bak tetramer and the thiols of these residues can be in closer proximity (Fig. 1d). Thus, upon oxidation by copper(II)(1,10-phenanthroline)_3_ reagent, two disulfide bonds will be formed between the cysteine residues (*i.e.*, for 69C′/111C and 69C/111C′) due to the symmetric nature of BGH (Fig. 2a). This will result in a Bak dimer with a shifted mobility in the denaturing polyacrylamide gel electrophoresis (PAGE) as previously shown in human Bak by Dewson *et al*.^24^. This was indeed the case (lane 8, Fig. 2f). The mobility shift was observed only when Bak was activated by p7/p15 Bid (lanes 7 and 8, Fig. 2f), proving the proximity of the cysteines only in the activated Bak but not in the inactive Bak. In contrast, the disulfide bond was not formed significantly in Bak mutant proteins containing only one cysteine at residue 69 or 111 (lanes 1 and 2, and 3 and 4, respectively, Fig. 2f), regardless of Bak activation by p7/p15 Bid. This further supports that the gel shift in lane 8 was due to the disulfide formation between cysteines at residues 69 and 111, which can be reduced under a reducing condition (Supplementary Information Figure S1c). Collectively, these results confirm that the BGH structure was formed in mitochondrial membrane by mouse Bak when it was activated by p7/p15 Bid, which is consistent with our previous *in vitro* data^27^ and with Dewson *et al*.^24^.

When an additional cysteine residue such as 143C (the penultimate C-terminal residue of α5 helix) was present in Bak 69C/111C mutant (*i.e.*, in Bak 69C/111C/143C), large oligomers of even numbered Bak proteins were formed upon oxidation after activation with p7/p15 Bid (lane 10, Fig. 2f; also see Supplementary Information Figure S1c). This was not observed in the absence of Bak activation (lane 9, Fig. 2f), indicating that 143C was brought to the oligomerization interface only when Bak was activated. Consistent with this, a dimer was formed in Bak 143C mutant in a p7/p15 Bid-dependent manner (lanes 5 and 6, Fig. 2f). These results showed that 143C was brought close to each other between BGHs in the Bak oligomeric pore, but not within a single BGH since the two 143C residues in a Bak BGH (*i.e.,* 143C and 143C′ in Fig. 2a) are separated too far away for disulfide formation (~50 Å between C_α_ atoms). In conclusion, the above results showed that the C-termini of α5 helices around residue 143 in the BGHs were near the Bak oligomerization interface. Likewise, even-numbered high order oligomers were formed in a p7/p15 Bid-dependent manner only in Bak 69C/111C/96C but not in Bak 96C (Fig. 2g; lanes 5 and 6, and lanes 1 and 2, respectively). The large distance between two 96C residues in a BGH (~45 Å between C_α_ atoms) also precludes the possibility of disulfide bond formation within a BGH. Thus, these results indicated that residue 96C, *i.e.*, the C-termini of α3 helices, were juxtaposed in the oligomeric Bak between neighboring BGHs. Finally, similar results were also observed in Bak 162C and Bak 69C/111C/162C (lanes 3, 4, 7 and 8), indicating that residue 162C, the penultimate C-terminal residue of helix α6, was also at the oligomerization interface in Bak pore, consistent with the formerly known ‘α6:α6 interface’^23^.

Additionally, the monomers of Bak 86C were cross-linked upon activation by p7/p15 Bid, consistent with the proximity of the two symmetry-related 86C residues in the BGH structure (86C and 86C′; ~10 Å or ~13 Å between C_β_ or C_α_ atoms, respectively) (Fig. 2h). This also indicated that the BGH structure was preserved in the Bak oligomeric pores in membrane.

The above results collectively showed that, in addition to α6 helices, the C-termini of helices α3 and α5 were juxtaposed between BGHs in oligomeric Bak (Fig. 2a) in apoptotic mitochondria, thus demonstrating the existence of the ‘α3/α5 interface.’ This is consistent with our earlier *in vitro* results obtained with recombinant mutant Bak proteins in liposomes^27^.

### EPR data further support the existence of ‘α3/α5 interface’ in oligomeric Bak pore

**Figure 3:**
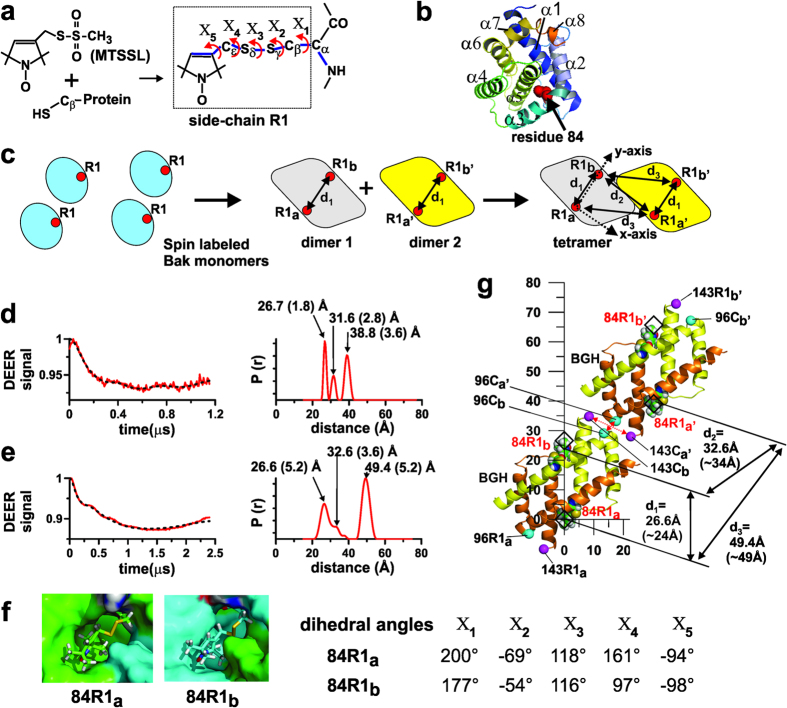
Inter-spin distances determined by double electron electron resonance (DEER) method. (**a**) Site-directed spin labeling (SDSL) reaction. A methanethiosulfonate spin label reacts with a cysteine residue, forming R1 side chain. The dihedral angles are measured counterclockwise, viewed through the indicated central bonds with the eclipse of the proximal 1^st^ and the distal 3^rd^ bonds (shown in thick blue lines) defined as 0°^ ^60,61. (**b**) The asparagine residue 84 (N84) in α2–3 loop selected for SDSL in Bak with its side chain shown in red spheres in a homology model^33^ of a soluble monomeric Bak, sBak-ΔC-His (residues 16–184). (**c**) When a singly spin labeled Bak forms an oligomeric pore via homodimer formation, various intra- and inter-dimer spin-spin distances (d_1_, etc.) can be determined by the DEER method. (**d**,**e**) X- (**d**) and Q-band (**e**) DEER data for oligomeric Bak formed with sBak-ΔC-His spin labeled at residue 84 (Bak/84R1) were obtained (red traces, left panel; black dotted lines are theoretical fit) and analyzed with DeerAnalysis2013^37^, resulting in the indicated most probable distances (Å) (width at half-height in parenthesis). (**f**) Rotamers of 84R1, 84R1_a_ and 84R1_b_, on the polypeptide chains A and B, in the hydrophobic pockets of BGH and their dihedral angles X_1_-X_5_. The error range of the dihedral angles is ~20°. The carbons in 84R1_a_ and 84R1_b_ are in green and cyan, respectively. The white, red, blue and yellow spheres represent hydrogens, oxygens, nitrogens and sulfurs, respectively. The images were generated using Pymol^59^. (**g**) Positions of 84R1s, ***a**, **b**, **a’***, and ***b***′ were calculated by 2-dimensional triangulation using the distances d_1_, d_2_, and d_3_ from (**e**), which are (0, 0), (0, 26.6), (30.1, 39.2), and (30.1, 65.8), respectively, in a xy-coordinate scaled in Å (See Supplementary Information Figure S2d for details). The inter-nitrogen line between the NO moieties of the 84R1_a_ and 84R1_b_ in BGH in (**f**) was superimposed to the ***a***-***b***, and ***a’***-***b’*** lines (black diamonds), respectively. Shown in the parentheses under d_1_, d_2_, and d_3_ are the corresponding distances from the resulting tetramer model. Note the proximity of the indicated amino acid pairs: 96C_a’_-96C_b_ and 143C_a’_-143C_b_ (C_α_-atoms shown in spheres).

Using site-directed spin labeling (SDSL) method (Fig. 3a), the inter-spin distances in the range of 15-80 Å can be measured by the double electron electron resonance (DEER) method^36^. There would be multiple spin-spin interactions between BGHs as well as within a BGH if spin-labeled Bak monomers formed oligomeric Bak pores (Fig. 3b,c). This was indeed the case in the Bak oligomeric pores formed with Bak/84R1, a Bak monomer spin labeled at residue 84 (Fig. 3d,e; also see Supplementary Information Figure S2). Clearly, three well-resolved peaks were observed in the probability vs. distance function obtained from the X-band DEER data using DeerAnalysis2013 program^37^ (Fig. 3d). Due to the short phase memory time of the electronic spins of the nitroxide labels in X-band experiment, the evolution time was limited and thus the accuracy of the longer distance was compromised. The Q-band DEER was thus used to overcome this. As shown in Fig. 3e (left panel), the evolution time was doubled and the distances over a longer range could be measured, which also revealed three distinct probability peaks when analyzed by DeerAnalysis2013 program (right panel) (also see Supplementary Information Figure S3c, 4th row). The probability distribution peak at ~27 Å (with the width of ~5 Å at half-height) was assigned to the intra-dimer spin pair and the other two peaks at ~33 Å and ~49 Å were assigned to the inter-dimer spin pairs (Fig. 3f). This was based on the control experiments shown in Supplementary Information Figure S3. When 84R1 residues were present in the BGHs of His-GFP-Bak tetramer in solution (Supplementary Information Figure S3a), only a single peak at ~27 Å was observed in the short distance range (Supplementary Information Figure S3c, 2nd and 3rd rows), indicating that this peak corresponds to the intra-BGH 84R1-84R1’ distance, consistent with modeling of the spin label rotamers in a BGH (see Supplementary Information Figure S3d,e).

If the structure of 84R1 in BGH is known, a 2-dimensional modeling of the two neighboring BGHs can be done by triangulation with the three distances determined above (Supplementary Information Figure S2d). The tetrameric GFP-Bak spin labeled at residue 84C could not be crystallized. We thus attempted to model the rotamers of 84R1 *in silico,* based on its low solvent accessibility (Supplementary Information Figure S2b) and low mobility (Supplementary Information Figures S2c and S4e,f) in oligomeric Bak in membrane. Modeling with MMM 2010 program^38^ did not readily sample such conformations that are consistent with the above observations (Supplementary Information Figure S3d). When the amino acid side chains around 84R1s were rearranged, hydrophobic pockets could be created on the surface of BGH (see Supplementary Information Figure S3e for details). Into these, rotamers of 84R1 could be placed in such a way that their N-O moieties are sequestered from the protein surface and the rotation of the nitroxide rings is inhibited (Fig. 3f, left panel). Considering the X_1_ and X_2_ dihedral angles of these rotamers, they correspond to the {t,m} rotamers^39^. The inter-spin distance (between the nitrogen atoms of the NO groups of 84R1 and 84R1’) in the BGH was ~24 Å. This is close to the measured distance, 26.6 (5.2) Å, within the range of the probability peak (Fig. 3e). When the 84R1-84R1’ inter-spin vector associated with the BGH was superimposed to the calculated inter-spin vectors from the triangulated points of R1s (Supplementary Information Figure S2d), the C-termini of α3 and α5 helices, specifically, residues 96Cs and 143Cs, could be brought close to each other surprisingly (with the C_β_-C_β_ distance of 6.5 Å and 14 Å, respectively) (Fig. 3g), consistent with the current cross-linking data (Fig. 2) and with the short inter-spin distances between 96R1s and 143R1s, respectively, as reported earlier^27^.

### The BGHs and helix α6 are adsorbed to the membrane surface at shallow depths

**Figure 4:**
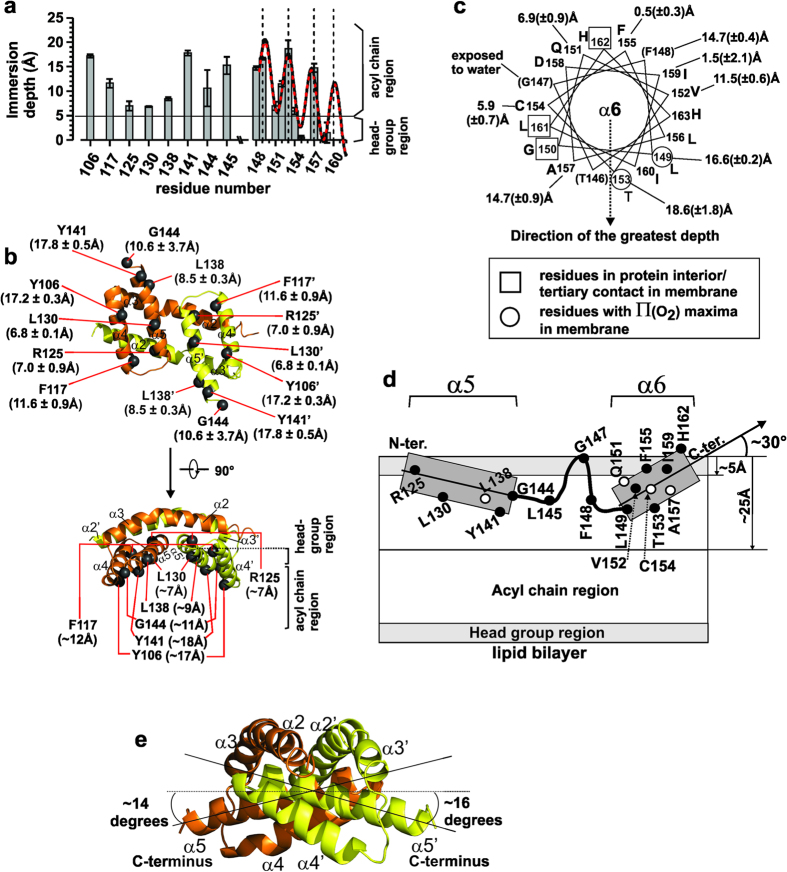
Interaction of BH3-in-groove homodimer and α6 helix with membrane. (**a**) Membrane immersion depths of the nitroxide spin label side chains (R1s) in mouse Bak BGH and α6 helix domains in oligomeric Bak are shown as a function of residue locations (average values of 2–3 experiments with error ranges indicated). The sinusoidal curves represent the depth-fitting curves for residues 149–158 with (solid) or without (dotted) residue 157 (see Supplementary Information Figure S6c for details). The residues marked with dotted vertical lines correspond to the local maxima in depth. (**b**) The immersion depths of R1s in the hydrophobic surface of BGH in top (top) and side (bottom) views. Black spheres represent C_α_-atoms of R1s. (**c**) Immersion depths and topological locations α6 residues in Bak in a helical wheel diagram. The direction of the greatest depth (see Supplementary Information Figure S6c) corresponds to the rotational orientation of the helix facing the membrane. The residues with a square mark correspond to those in tertiary contacts or in protein interior. The circled residues represent amino acid locations at which the accessibility parameter to oxygen, ∏(O_2_), reaches a local maximum in each helical turn (see Supplementary Information Figure S6a). (**d**) Helix tilting angle and the topological locations of the indicated R1s in α5-α6 region in oligomeric Bak are shown. Approximate C_α_-positions of the R1s in α5 (residues 123–144), α5-6 loop (residues 145–148) and α6 (residues 149–163) are shown relative to the membrane. Helix α6 was tilted toward the N-terminus by ~30° by the depth-fitting analysis (see Supplementary Information Figure S6c). (**e**) The tilting angles of α5 helices in mouse BGH are shown relative to a hypothetical horizontal plane (dotted line). See also Supplemental Figures S5 and S6.

To better define how Bak homodimers interact with the membrane, we measured the membrane immersion depths of selected residues in BGH and helix α6 using SDSL (Fig. 4a, S5 and S6).

Residues Y106 and F117 are located on helix α4 on the hydrophobic surface of BGH (Fig. 4b). Their corresponding R1 sidechains were located at the depths of ~17 Å and ~12 Å from the membrane surface, respectively (Fig. 4a,b). The immersion depths of α5 residues on the hydrophobic surface such as 130R1, 138R1, 141R1 and 144R1 were ~7 Å, ~9 Å, ~18 Å, ~11 Å, respectively, indicating that these residues were also located in the hydrocarbon region of the lipid bilayer (Fig. 4a,b). Residue 125R1 located in the first helical turn of α5 helix also had a depth of ~7 Å, indicating that the N-terminus of α5 was also in contact with the membrane. These results collectively showed that the hydrophobic surface of BGH is in contact with the membrane.

In the α5–6 loop (residues 145-148), residue 147R1 was water-exposed while others such as 145R1 and 148R1 were buried at ~15 Å depth from the membrane surface (Fig. 4c), indicating that the loop was partially exposed to water (Fig. 4d; also see Supplementary Information Figure S6b legend).

Residues on helix α6 were also interacting with the membrane as summarized in Fig. 4a,c,d. The immersion depths of R1s in helix α6 (residues 149–163) had an oscillating pattern as a function of residue locations (Fig. 4a,c). The depths could be best-fitted with an α-helix inserted into the membrane with its helical axis tilted toward the N-terminus at an angle of ~30° (Fig. 4a,d, and S6c). The direction of the greatest depth was very close to the radial line for residue 153R1 in the helical wheel diagram for α6 helix (Fig. 4c, dotted vertical arrow). In this state, residues 149R1, 151R1, 152R1, 153R1 and 157R1 were located in the acyl chain region of the lipid bilayer (white region in Fig. 4d) while residues 155R1 and 159R1 were in the head group region (gray region in Fig. 4d) (Also see Fig. 4a). Residues 150R1, 161R1 and 162R1 had immobile EPR lineshapes (Supplementary Information Figure S5d) with small accessibility parameters for oxygen and (Supplementary Information Figure S6), indicating that these residues are either in protein interior or in tertiary contact (Supplementary Information Figure S6b).

Noting that helix α6 was a surface-adsorbed helix and that the α5-6 loop was also partially exposed to water, the pattern in the depths of α5 residues could be best explained by a surface-adsorbed helix with its axis tilted toward the C-terminus (Fig. 4d,e). These results, along with the depths of residues in α4 helix (106R1 and 117R1), showed that the BGH and the α6 helices in the Bak homodimer were adsorbed to the membrane surface, consistent with Westphal *et al*.^30^.

## Discussion

The X-ray crystal structure of the mouse BGHs determined here has the characteristic binding pocket formed by helices α2-α5, to which the BH3 domain in the extended α2-α3 helix of its symmetry-related partner is bound^25^,^29^ (Fig. 1d–f). As a result, a raft-like structure of two-layers of α-helices is formed as is also seen in other BGHs formed by human Bak and Bax (Fig. 1e,f). The surface formed by a pair of α4-α5 helices is hydrophobic and curved, suited for interaction with the membrane (Figs 1e and 4). The chemical cross-linking data from Bak 86C and Bak 69C/111C in apoptotic mitochondria (Fig. 2) were consistent with the BGH structure determined here (Fig. 1). The EPR spectra of spin-labeled residues attached to various locations of the BGH were very similar whether they were present in the tetrameric GFP-Bak in solution or in oligomeric Bak in membrane (Supplementary Information Figure S4f). Also, the distance between 84R1s within a BGH domain remained essentially the same in the above two states (Supplementary Information Figure S3c). All these strongly suggest that the BGH structure in the oligomeric Bak pore in the membrane is very similar to the X-ray crystal structure of BGH observed in solution state, consistent with our previous report^27^.

In the GFP-Bak tetramer, the two BGH units form a partly open hydrophobic pocket in which the hydrophobic surfaces are sequestered away from the surface and thus not readily available for interaction with the membrane (Fig.1d). Furthermore, between the two BGHs, the C-terminal residues of the two closer α3 helices are separated at a large distance (~40 Å) unlike what was observed in the membrane (Fig. 2). Thus, the ‘α3/α5 interface’ was implicated neither in the GFP-Bak tetramer nor in the crystal contacts (Supplementary Information Figure S1b).

The immersion depths of the R1s in oligomeric Bak indicated that the BGH and α6 helices are adsorbed to the membrane surface at shallow depths (Fig. 4), consistent with others^30^. In our BGH structure, the two central α5 helices in the BGH form an angle of approximately 15 (±1) degrees relative to a hypothetical horizontal plane that is set parallel to the α2-α3 helices (Fig. 4e). Assuming that BGH is immersed flat in the membrane, the helical tilt of α5 would be approximately 15 (±1) degrees relative to the membrane surface. The membrane-immersion depths of 130R1, 138R1, 141R1 and 144R1 in α5 helix appear to be consistent with this assumption (Fig. 4d,e). Note that the immersion depth of a R1 side chain depends not only on the position of its α-carbon (C_α_) but also on the side chain’s direction relative to the membrane normal vector. For this reason, pairs of residues such as 130R1 and 138R1, 106R1 on α4 and 141R1 on α5 had similar depths despite the differences in the depths of the C_α_ atoms (Fig. 4b).

The chemical cross-linking results clearly demonstrated the proximity of the C-termini of α3 and α5 helices between neighboring homodimers in the Bak oligomeric pore formed in the mitochondrial outer membrane (Fig. 2a,f,g), confirming our *in vitro* study^27^ and its biological relevance. Very recently, similar results were also observed in oligomeric Bax^28^, indicating that this ‘α3/α5 interface’ is common both in Bak and Bax oligomeric pores. The DEER results also support the existence of this interface (Fig. 3g).

Recently, Westphal *et al*. proposed a model of lipidic pore formed by apoptotic Bak oligomers^30^. In this model, Bak BGHs and α9 helices were assumed to remain on the flat region of the membrane while the helical hairpin, formed by α6 and α7-α8 extended helices, was hypothesized to line the central lumen of the lipidic pore in a transmembrane orientation, reaching well beyond the core of the membrane. However, our molecular modeling indicated that the α6-α8 helical hairpin with the extended length of ~30 Å, if it existed, is too short to reach beyond the midpoint of a lipidic pore when it is adsorbed to the surface of a lipidic pore formed in a ~45–50 Å-thick lipid bilayer. Furthermore, if the hypothesized α6-α8 helical hairpin existed on the surface of the lipidic pore lumen, parallel arrangement of the hairpins within the pore lumen would make it difficult for α6 helices to make direct contacts between them, contrary to the cross-linking result with Bak/162C (Fig. 2g) and the short inter-spin distance between 162R1-162R1,’ which is ~5-12 Å^27^.

Based on the nitroxide inter-spin distances in Bax, Bleicken *et al*.^32^ proposed an alternative model of Bax lipidic pore, where the Bax homodimers ‘clamp’ the toroidal surface of the lipidic pore as mentioned in the Introduction. They assumed that the transmembrane orientation of helix α9 alternates in the membrane. However, it was suggested that α9 helices are associated in a parallel transmembrane (TM) orientation in Bax apoptotic pores^28^,^40^. Iyer *et al*. also suggested that the ‘α9:α9 interface’ in Bak pore is formed by parallel association of α9 helices in a transmembrane orientation^31^. Thus, it’s difficult, if not impossible, to envision that the TM helix of Bax or Bak will switch its orientation during pore formation. Zhang *et al*. recently suggested that Bax α9 helices line the large lipidic pores formed by Bax^28^. In case of Bak, a TM sequence was not essential in pore formation^33^ and its direct contribution to the pore structure was not supported experimentally^31^.

**Figure 5:**
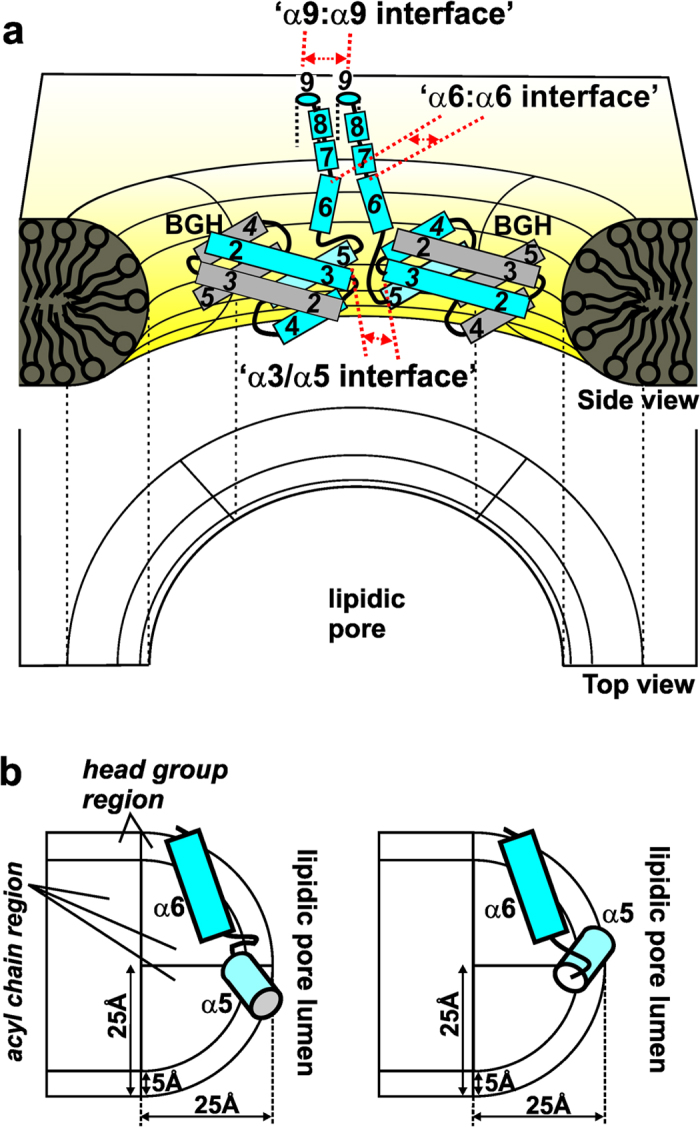
Schematic model of a lipidic pore formed by Bak homodimers. (**a**) The BGHs line the lumen of the lipidic pore. Helices α6-α8 are adsorbed to the toroidal surface of the pore and anchored to the transmembrane helix α9 that stays in the flat region of the membrane around the pore. The three separate oligomerization interfaces are indicated. Numbered rectangles are the corresponding helices in Bak (Helices α6-α9 were omitted for two gray-colored Bak molecules and helix α1 omitted for all for clarity.). Of note, the BGHs may exist close to the flat region/toroidal surface boundary of the lipidic pore. (**b**) Two possible arrangements of α5-α6 domains are shown in a cross-sectional side view of a schematic Bak lipidic pore. These correspond to the two possible orientations of α5 helices (in cyan) in a BGH that is adsorbed onto the toroidal surface (See the two α5-α6 domains in two separate BGHs shown in blue and cyan in (**a**)).

Now, a more detailed working model of the Bak lipidic pore, built on our previous one^27^, is proposed to resolve the above issues (Fig. 5a). Here, the TM α9 helices are hypothesized to interact with each other, forming the ‘α9:α9 interface’ within the flat region of the membrane around the lipidic pore as suggested by others^28^,^31^,^40^. The extended α6-α8 helices, while being adsorbed to the lipidic pore lumen, are hypothesized to be in a pseudo-parallel orientation^27^, tethering the BGHs to α9 helices. This forms the ‘α6:α6 interfaces’ between the neighboring Bak homodimers, consistent with our and others’ results^23^,^27^,^41^. In this model, the topological arrangements of α5-α6 helical hairpins on the lipidic pore surface can also be explained as schematically shown in Fig. 5b. When a BGH is adsorbed within the curved surface of the lipidic pore as depicted in Fig. 5a, the α5-α6 helical segment is inserted into the membrane with their helical axes tilted toward the C- and N-terminus, respectively (Fig. 5b), consistent with the experimental results (Fig. 4d). Note that the two possible arrangements of the α5-α6 helical hairpins in Fig. 5b correspond to the two conformations of each monomer in the BGH adsorbed to the lipidic pore (Fig. 5a). Also note that the C-termini of α3 and α5 helices can be brought to a close proximity between BGHs of any neighboring Bak homodimers, which are presumably on the curved surface of the lipidic pore, forming the ‘α3/α5 interface’ (Fig. 5a). Currently, the exact location of BGHs within the lipidic pore is not known^18^,^19^,^21^. The dimension of a BGH is approximately 40 Å from top to bottom when oriented as depicted in Fig. 5a, which can be placed within the lipidic pore. The topology of Bax pores or arcs recently determined by the atomic force microscopy (AFM)^18^ showed a protrusion of about 40 (±10) Å at the rim relative to the membrane surface. Earlier, Epand *et al*. reported a protrusion of 24 Å at the pore rim in an AFM image of Bax pore with the diameter of ~20 nm^42^. These suggest that BGHs and/or other domains (*e.g.*, helices α1 or α6-α9) of Bax or Bak might cover both the flat region and the toroidal surface of the lipidic pore. Finally, noting that a simple 2-dimensional triangulation with the measured intra- and inter-dimer distances could explain the proximity of residues in the ‘α3/α5 interface’ (Fig. 3), the diameters of the lipidic pores formed by Bak in liposomes in this study should be very large, which is consistent with other’s reports^18^,^19^,^20^,^21^,^42^,^43^.

In conclusion, we have determined the mouse Bak BGH structure, which allowed more accurate modeling of the DEER distances observed within and between the BGHs. The curved hydrophobic surface of the BGH was immersed in the membrane at a shallow depth. The BGHs were shown to oligomerize via the ‘α3/α5 interface’ in mitochondria. These findings led us to propose a probable assembly of the Bak homodimers in the mitochondrial apoptotic pore. This sheds important insights into the action mechanism of Bak or Bax in mitochondrial apoptosis pathway. However, the function of the novel ‘α3/α5 interface’ in Bak oligomerization and pore formation is unknown and it requires further investigation.

## Methods

### X-ray crystallography

#### Protein expression and purification

A DNA fragment for α2-α5 of mouse BAK (residues 66-144) was subcloned into the pET-28a-GFP A206N vector (designated as pYEGFP_A206N _BAK_H2-H5_pET28a)^25^,^44^. The resulting vector expresses an amino (N)-terminally hexahistidine tagged green fluorescent protein (with A206N mutation) fused to mouse Bak α2-α5 helices (designated as His-GFP-Bak) in *E. coli* BL21 (DE3) (see Fig. 1a). The N-terminal sequence of His-tag and the thrombin cleavage sequence is MGSSHHHHHHSSGLVPR/GSH in a single letter amino acid code (thrombin recognition sequence and cleavage site indicated by an underline and ‘/’, respectively). Cells were grown to the OD_600nm_ of ~1.2 at 37 °C in Super Broth and were induced to express the fusion protein with 1mM IPTG for 15 hrs at 18 °C. Cells were centrifuged at 4,700 g and the cell pellets were resuspended in TBS (20 mM Tris pH 8.0, 150  mM NaCl) buffer containing 10  mM imidazole. The cells were treated with lysozyme, freeze-thawed and lysed by sonication. The protein was purified with Ni-NTA metal affinity chromatography, followed by N-terminal his tag removal by thrombin cleavage. The tag-less GFP-Bak was purified by another round of Ni-NTA metal affinity chromatography in TBS buffer. The protein was concentrated to a final concentration of ~20 mg/ml using Amicon Ultra concentrator with the molecular weight cutoff of 50 kDa (Millipore).

#### Crystallization of GFP-Bak, data collection and structure determination

Purified GFP-Bak was crystallized by hanging-drop vapor diffusion method adapting Czabotar *et al*.^25^ as described in the Supplementary Information. Diffraction data were collected at the GM/CA-CAT, Advanced Photon Sources in Argonne National Laboratory and the structure was determined by molecular replacement as described in detail in the Supplementary Information (also see Table 1).

### Chemical cross-linking experiments

#### Cell culture

The wild type and *bax*^*−/−*^ *bak*^*−/−*^ double knockout mouse embryonic fibroblasts (MEFs)^45^ were obtained from the laboratory of Stanley Korsmeyer (Dana-Farber Cancer Institute, Boston, MA). MEFs were cultured on cell culture dishes (Genesee, San Diego, CA) in Dulbecco’s modified Eagle’s medium (DMEM) (Invitrogen, Carlsbad, CA) supplemented with 10% heat-inactivated fetal bovine serum, 2 mM L-glutamine, 100 μM nonessential amino acids (NEAA), 1% penicillin/streptomycin at 37 °C in a 5% CO_2_ incubator.

#### Preparation of MEF cell lines expressing mutant Bak protein

Retroviral plasmids harboring genes for single, double, or triple cysteine substitution BAK mutant proteins were prepared with the template plasmid pMSCV-mBAK-IRES-GFP^46^ using the QuikChange Site-directed mutagenesis kit (Stratagene) according to the manufacturer’s instructions. Retroviral preparations were made by cotransfecting the ecotropic 293 cells with pMSCV-mBAK-IRES-GFP vector, pECO and pSG5-BCLx using Lipofectamine 2000 (Invitrogen). After 72 hrs of transfection, the cell culture medium containing the virus was collected and cleared by centrifugation at 3,000 g for 10 min at room temperature. The viral supernatant was then used to infect the MEF cells derived from *bax*^*−/−*^ *bak*^*−/−*^double knockout mice^47^. MEF cells stably expressing the Bak proteins were selected by serial passages (minimum 3) in the presence of puromycin (2 μg/ml) in the above cell culture medium for 7 days on cell culture flasks (Genesee, San Diego, CA).

#### Isolation of mitochondria

For each Bak mutant, puromycin-selected cells above were expanded and plated onto 4 culture dishes (15 cm in diameter, Genesee, CA) in the selection medium. The cells were harvested by scraping and the mitochondria were isolated at 4 °C from these cells using a mitochondria isolation kit (Thermo Scientific) according to the manufacturer’s instructions. Cells, resuspended in the resuspension buffer in the kit, were disrupted by 10 passages through a 21 G syringe needle. Heavy membrane fractions were removed by two consecutive centrifugations at 700 g for 10 min at 4 °C. Mitochondrial fractions were pelleted by centrifuging the resulting supernatant at 12,000 g for 15 min. The resulting pellets were gently resuspended in a trehalose buffer (300 mM trehalose, 10 mM KCl, 1 mM EGTA, 10 mM HEPES, pH 7.4) to a final protein concentration of ~2 mg/ml. The protein concentration was determined using Pierce™ BCA Protein Assay Kit (Thermo scientific).

#### Cytochrome *c* release assay

Mitochondria (60 μg in protein quantity) were spun down at 12,000 g for 10 min at 4 °C. They were resuspended in 100 μl of the cytochrome *c* release assay buffer (20 mM HEPES/KOH pH 7.5, 100 mM sucrose, 80 mM KCl, 1 mM ATP, 80 μM ADP, 5 mM Na Succinate, 1 mM DL-dithiothreitol (DTT)) in the presence of 0 or 100 nM p7/p15 Bid, and further incubated for 30 min at 30 °C. A volume of 50 μl of the reaction mixture was set aside on ice for the cross-linking experiments below. Cytochrome *c* released into the medium was collected by centrifuging the remaining samples at 12,000 g for 10 min at 4 °C. The resulting pellet was resupended in the assay buffer (50 μl). A volume of 10 μl of 6x SDS sample buffer (0.375 M Tris pH 6.8, 12% (w/v) SDS, 60% (v/v) glycerol, 0.6 M DTT, 0.06% (w/v) bromophenol blue) was mixed with 50 μl of the resulting supernatant and resuspended mitochondrial samples. One sixth of each paired sample was subjected to SDS-PAGE under a reducing condition, followed by immunoblotting. The primary and the secondary antibodies used were mouse monoclonal anti-cytochrome *c* antibody (Santa Cruz, Cat. # sc-13156)/Anti-rabbit IgG (Perkin Elmer, Cat. # NEF812001EA). The percentage of released cytochrome *c* was determined by measuring the intensities of the Western blotting images using ImageJ software.

#### Disulfide cross-linking experiment

First, a necessary volume (*e.g.*, 1 μl) of copper(II)(1,10-phenanthroline)_3_ (CuPhe) solution (150 mM Copper sulfate (Sigma), 500 mM 1,10-phenanthroline (Sigma) in 20%(v/v) ethanol) was freshly diluted 100-fold into the cross-linking buffer (*e.g.*, 1 ml 20 mM HEPES/KOH pH 7.5, 150 mM KCl, 100 mM sucrose, 5 mM MgCl_2_, 2 mM NaAsO_2_)^35^. The mitochondrial samples (containing 30 μg mitochondrial proteins) set aside above were centrifuged at 12,000 g for 10 min at 4 °C. The resulting pellets were resuspended in a volume of 20 μl cross-linking buffer made above and were then further incubated for 30 min on ice. The reaction was quenched by mixing the reaction mixture with an equal volume of 2x nonreducing SDS-sample buffer containing 10 mM EDTA. Samples were analyzed by SDS-PAGE, followed by immunoblotting. The primary and the secondary antibodies used were rabbit polyclonal anti-BAK aa23–38 antibody (Millipore, Cat. # 06–536) and HRP-conjugated goat anti-mouse antibody (Santa Cruz, Cat. # sc-2062).

### Site-directed spin labeling experiments

#### Protein preparation

The cysteine substitution mutant proteins of the C-terminally hexahistidine-tagged soluble form of the mouse Bak proteins (residues 16–184 of the full length protein with a C154S amino acid substitution, designated as sBak-ΔC-His) were prepared and spin labeled with (1-oxyl-2,2,5,5,-tetramethyl-Δ3-pyroline-3-methyl) methanethiosulfonate spin label (MTSSL) (Toronto Research Chemicals, Inc., Toronto, Canada) as described^33^ (Also see the Supplementary Information). N-terminally hexahistidine-tagged p7/p15Bid (designated as p7/p15 Bid) was prepared as described^48^,^49^.

#### Liposome preparation

Large unilamellar vesicles (LUVs) mimicking the lipid composition of mitochondrial contact sites were made as described (See Supplementary Information). LUVs encapsulating fluorescein isothiocyanate-dextran 10 (FITC-dextran, 10 kDa, Invitrogen) were prepared with the same lipid composition and stored in the presence of 18% (v/v) glycerol as described^33^.

#### Liposome dye release assay

Dye release experiments were carried out in buffer A (20 mM HEPES, 150 mM KCl, pH 7.0) with spin labeled sBak-ΔC-His proteins (5 nM) in the presence of 25 nM p7/p15 Bid with LUVs (10 μg/ml lipids) encapsulating FITC-dextran (10 kDa) as described^27^ (See Supplementary Information for details).

#### Preparation of oligomeric Bak in membrane

Oligomeric Bak samples were prepared using the above LUVs in the presence of the activator protein p7/p15Bid with a mixture of the spin-labeled sBak-ΔC-His proteins and the unlabeled soluble Bak molecule (sBak/C154S-ΔC-His) at a ratio of 3:4 (for depth measurement) or 7:0 (for DEER experiment) as described^33^ (See Supplementary Information for details).

#### EPR spectroscopy

X-band continuous wave (CW) EPR experiments were carried out as follows. CW EPR spectra of the singly spin-labeled sBak-ΔC-His proteins (in 18% (v/v) glycerol) in solution or in membrane-inserted oligomeric BAK samples, were obtained on a Bruker EleXsys 580 spectrometer using a Bruker High Sensitivity resonator or a loop gap resonator (JAGMAR, Krakow, Poland)^50^ at 2-mW incident microwave power using a field modulation of 1.0–1.5 Gauss at 100 kHz at room temperature. Power saturation method was used to measure the accessibility parameters of air oxygen and NiEDDA (Nickel(II) ethylenediaminediacetate) (*i.e.*, ∏(O_2_) and ∏(NiEDDA) at 5 mM or 50 mM). The accessibility parameter of a R1 residue to a collision reagent is a quantity that is proportional to the collision frequency between the spin label and the collision reagent (*e.g.*, molecular air oxygen or Ni(II)ethylenediaminediacetate (NiEDDA)), which can be used to map the topological locations of proteins^51^. Samples in a volume of 3 μls were placed in a gas-permeable TPX capillary (Molecular Specialties, Inc., Milwaukee, WI) and the power saturation data were obtained by recording the central lines of the EPR spectra of the samples in the window of 15 Gauss over 0.4–100 milliwatts microwave incident power successively in the absence or presence of air oxygen or NiEDDA (5 mM or 50 mM) using the loop gap resonator^50^ as described^52^,^53^. Nitrogen gas (Nitrogen HP 99.995%, Specialty Gases of America, Inc., Toledo, OH) was used to flush the samples. Power saturation data were analyzed to calculate the P_1/2_ values using the R program (version 2.12.0)^54^ as described^48^. The accessibility parameters, ∏, were calculated as defined^52^,^53^; ∏ (x) = {(P_1/2_ (x)−P_1/2_°)/ΔH_pp_}/{P_1/2_(DPPH)/ΔH_pp_ (DPPH)}, where x = O_2_ or 5 mM (or 50 mM) NiEDDA; and P_1/2_° is the P_1/2_ value without any collision reagent under nitrogen gas; P_1/2_(DPPH) is the P_1/2_ value of the standard sample of crystalline 2,2-diphenyl-1-picrylhydrazyl (DPPH) in KCl; ΔH_pp_ and ΔH_pp_ (DPPH) are the peak-to-peak line widths of the sample’s and the DPPH’s EPR spectra, respectively. For depth measurement, the Φ value, which is the natural log of the ratio of ∏(O_2_) to ∏(50 mM NiEDDA) (i.e., log_*e*_ [∏(O_2_)/∏(50 mM NiEDDA)]) was determined for each R1 residue. The Φ value was converted to the membrane immersion depth using a Φ-depth calibration curve as reported^33^. The depth standards used were PC tempo, N-tempoylpalmitamide, 5-doxyl-PC, 7-doxyl PC, and 10-doxyl PC, for which the immersion depths were −5.0, 0.0, 8.1, 10.5 and 14.0 Å, respectively^55^. NiEDDA was synthesized as described^53^.

DEER experiments were done using the 4-pulse DEER sequence^56^ as described^27^. The X-band DEER experiments were carried out with an in-house Bruker EleXsys 580 spectrometer as described^27^. The Q-band DEER spectroscopy was carried out at the National Biomedical EPR Center, Milwaukee on a Q-band Bruker ELEXSYS 580 equipped with an EN5107D2 resonator and a 10 W amplifier at 80 K using a four-pulse sequence. Q-band DEER measurements were done after exchanging the sample buffer with deuterated buffers. First, the deuterated 20 mM Tris, 150 mM NaCl, pH 8 (TBS) buffer was prepared as follows; A quantity of 8.6 milligrams of Tris-HCl (FisherScientific), 5.5 milligrams of Tris base (FisherScientific), and 43.8 milligrams of NaCl (Sigma-Aldrich) were dissolved in a final volume of 5 ml deuterated water (D_2_O)(100%, Sigma-Aldrich). Deuterated buffer A was made by dissolving 23.8 milligrams of HEPES (Sigma-Aldrich), 55.9 milligrams of KCl (Sigma-Aldrich) into a final volume of 5 ml D_2_O and the pH was adjusted to 6.6, which is equal to pD 7.0^57^. Spin labeled His-GFP-Bak proteins prepared in TBS were buffer-exchanged in the above deuterated TBS by repeating two cycles of 10-fold dilution and centrifugal concentration in a concentrator (MWCO of 50 kDa). Oligomeric Bak samples prepared in membrane as described above in buffer A were resuspended in 100 μls of the deuterated buffer A. These were centrifuged at ~110,000 × g for 30 min at room temperature. The heavy buffer layer was removed by using a glass capillary. Finally, thus prepared buffer exchanged samples were mixed with deuterated glycerol (Sigma-Aldrich) to a final concentration of 18% (v/v) for cryoprotection, typically in 13 μl. Samples were contained in fire-sealed quartz capillaries (1.1 mm × 1.6 mm; VitroCom) and flash frozen in a dry ice and acetone mixture and loaded onto the spectrometer for DEER experiments. DEER data were analyzed with DeerAnalysis^37^ or DEFit program^58^.

## Additional Information

**Accession codes**: The atomic coordinates and the structure factors (PDB ID: 5KTG) have been deposited in the Protein Data Bank, Research Collaboratory for Structural Bioinformatics (http://www.rcsb.org/).

**How to cite this article**: Mandal, T. *et al*. Assembly of Bak homodimers into higher order homooligomers in the mitochondrial apoptotic pore. *Sci. Rep.* **6**, 30763; doi: 10.1038/srep30763 (2016).

## Supplementary Material

Supplementary Information

## Figures and Tables

**Table 1:** Statistics of the X-ray diffraction data.

Resolution range (Å)	50–2.8
Space group	P3_1_21
Unit cell (Å)	171.64, 171.64, 98.19
Unit cell (deg)	90, 90, 120
Wavelength (Å)	1.0
Beam lines	GM/CA-CAT, APS
Number of measurements	584081
Number of unique reflections	40122
Completeness of data (%)	
Overall	97.4
Last shell/resolution range (Å)	81.7 (2.9–2.8)
R_sym_ (%)	
Overall	15.1
Last shell/resolution range (Å)	77.7 (2.9–2.8)
I/sigma	
Overall	13.1
Last shell/resolution range (Å)	0.8 (2.9–2.8)
R_work_ (%)	24.36
R_free_ (%)	27.63
Mean B (Å^2^)	87.16
Root-mean-squared deviations	
Bonds (Å)	0.018
Angles (deg)	2.14
Ramachandran plot statistics (%)	
Most favored	89.9
Additional allowed	9.7
Generously allowed	0.4
Disallowed	0.0

*R*_*sym*_ = Σ_j_ Σ_i_|*I*_*ij*_ − <*I*_*j*_>| / Σ_i_ Σ_*j*_ *I*_*ij*_, where i runs over multiple observations of the same intensity, and j runs over all crystallographic unique intensities. R_factor_ = Σ||*F*_*obs*_| − |*F*_*calc*_||/Σ|*F*_*obs*_|. R_free_ was calculated with 5% of the reflections selected.
