# Supplementary material for: Assembly of Bak homodimers into higher order homooligomers in the mitochondrial apoptotic pore
Source: Sci Rep. 2016 Aug 4;6:30763. doi: 10.1038/srep30763 (PMC4973285; doi:10.1038/srep30763)
Supplement: Supplementary Information [file srep30763-s1.pdf]

Supplementary Information

Subjects: Biological Sciences/Cell biology/Cell death/Apoptosis

Title

Assembly of Bak homodimers into higher order homooligomers in the mitochondrial apoptotic pore

Authors/Affiliations

Tirtha Mandal[1], Seungjin Shin[1], Sreevidya Aluvila[1], Hui-Chen Chen[2], Carter Grieve[1], Jun-Yong Choe[1], Emily H. Cheng[2], Eric J. Hustedt[3], Kyoung Joon Oh[1*]

[1]Department of Biochemistry and Molecular Biology, Rosalind Franklin University of Medicine and Science, North Chicago, Illinois 60064, USA.

[2]Human Oncology and Pathogenesis Program, Memorial Sloan Kettering Cancer Center, New York, New York 10065, USA.

[3]Molecular Physiology and Biophysics, Vanderbilt University School of Medicine, Nashville, Tennessee 37232, USA.

*Correspondence and requests for materials should be addressed to K.J.O. (email: kyoung.oh@rosalindfranklin.edu)

Supplemental Figures

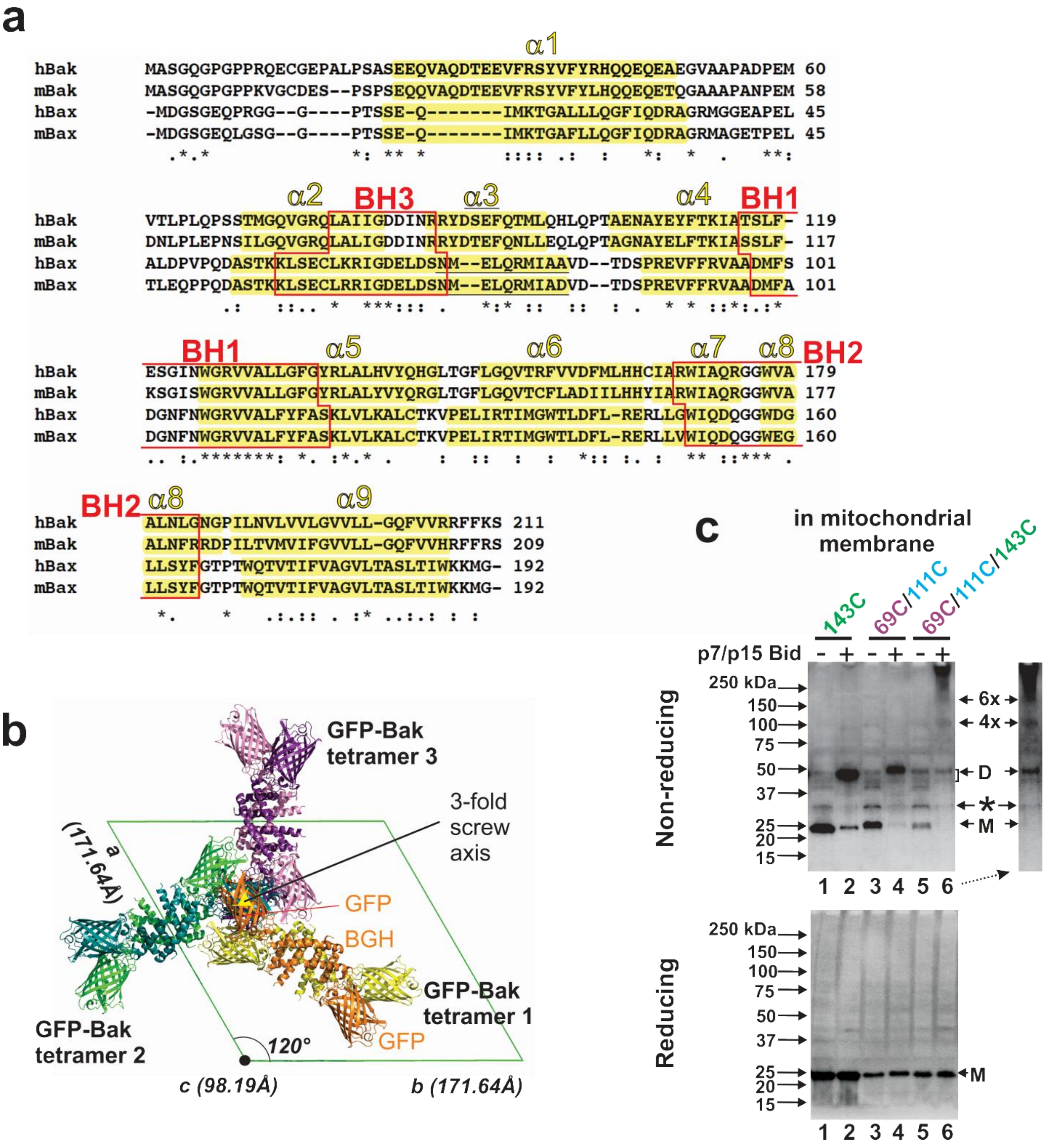

Figure S1.

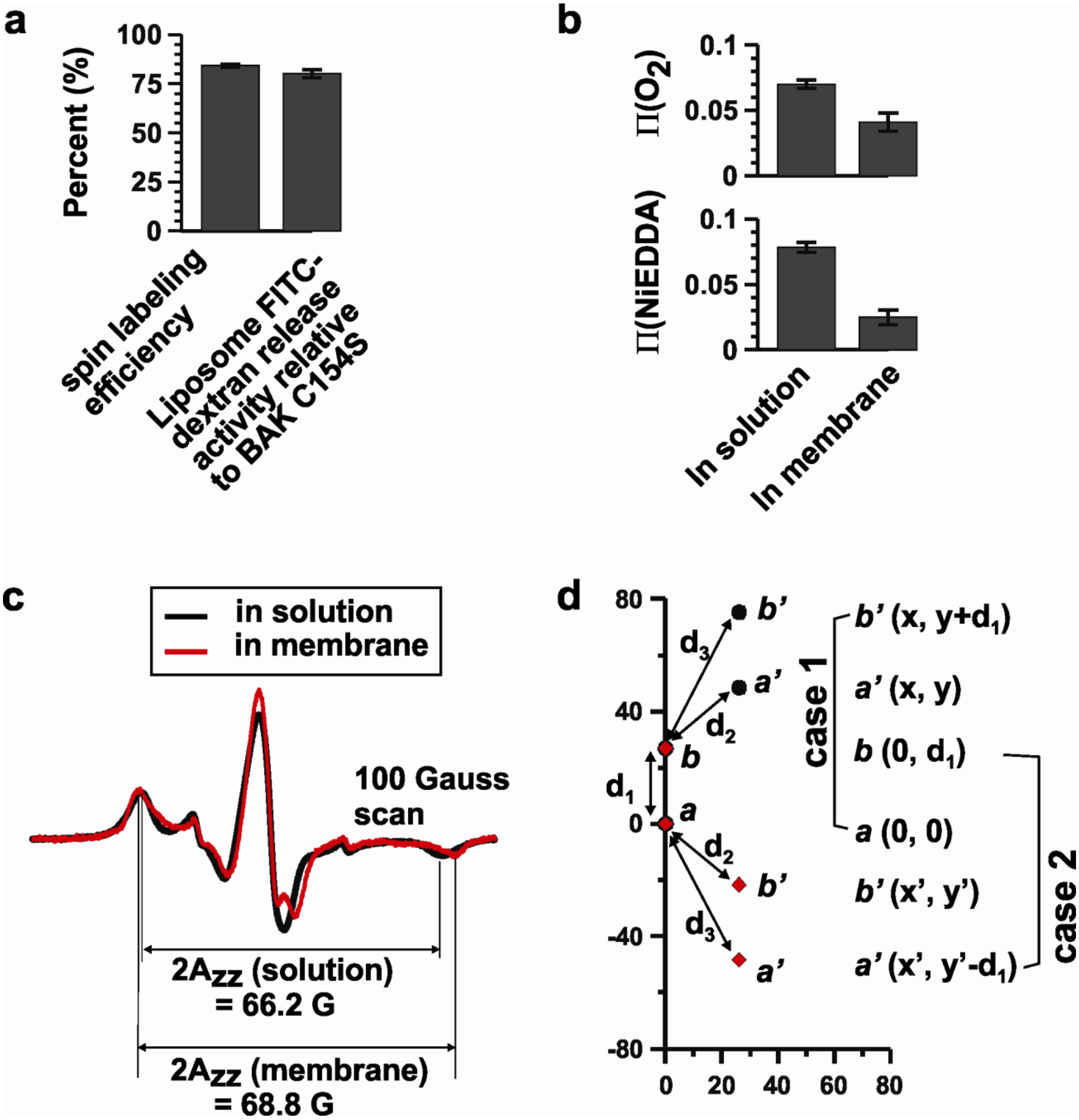
a
Percent (%)
100
75
50
25
0
spin labeling efficiency
Liposome FITC-dextran release activity relative to BAK C154S
b
Π(O2)
0.1
0.05
0
Π(NiEDDA)
0.1
0.05
0
In solution
In membrane
c
in solution
in membrane
100 Gauss scan
2Azz (solution) = 66.2 G
2Azz (membrane) = 68.8 G
d
80
40
0
-40
-80
0
20
40
60
80
d1
d2
d3
b'
a'
b
a
b' (x, y+d1)
a' (x, y)
b (0, d1)
a (0, 0)
b' (x', y')
a' (x', y'-d1)
case 1
case 2

Figure S2.

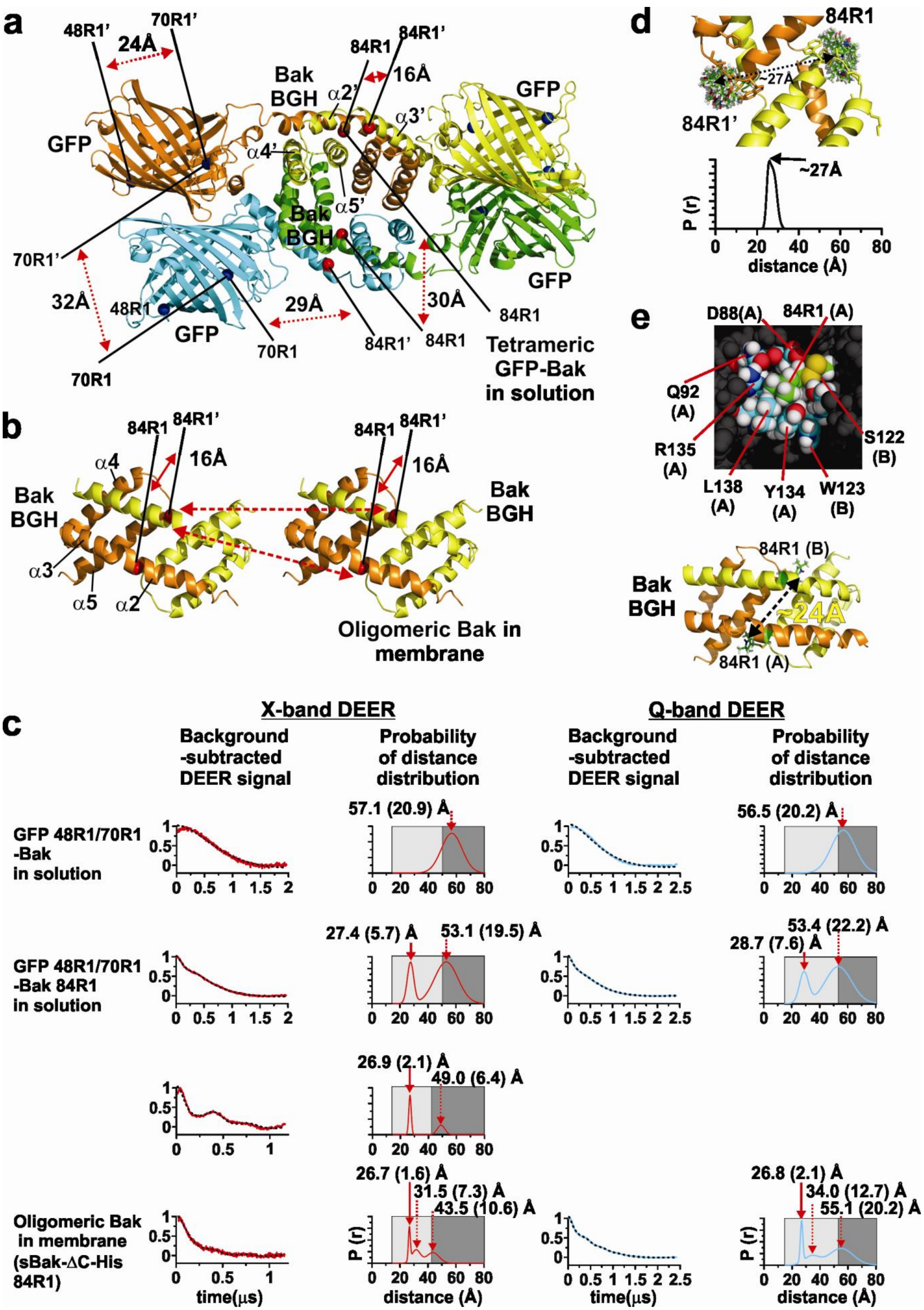
a
48R1'
70R1'
24Å
84R1
84R1'
16Å
Bak
BGH
α2'
α3'
GFP
GFP
α4'
α5'
Bak
BGH
70R1'
32Å
48R1
GFP
29Å
30Å
GFP
84R1
70R1
84R1'
84R1
Tetrameric
GFP-Bak
in solution
70R1
b
84R1
84R1'
16Å
84R1
84R1'
16Å
α4
Bak
BGH
Bak
BGH
α3
α5
α2
Oligomeric Bak in
membrane
d
84R1
~27Å
84R1'
~27Å
P (r)
distance (Å)
e
D88(A)
84R1 (A)
Q92
(A)
R135
(A)
S122
(B)
L138
(A)
Y134
(A)
W123
(B)
84R1 (B)
Bak
BGH
~24Å
84R1 (A)
c
X-band DEER
Q-band DEER
Background
-subtracted
DEER signal
Probability
of distance
distribution
Background
-subtracted
DEER signal
Probability
of distance
distribution
GFP 48R1/70R1
-Bak
in solution
57.1 (20.9) Å
56.5 (20.2) Å
GFP 48R1/70R1
-Bak 84R1
in solution
27.4 (5.7) Å
53.1 (19.5) Å
53.4 (22.2) Å
28.7 (7.6) Å
26.9 (2.1) Å
49.0 (6.4) Å
Oligomeric Bak
in membrane
(sBak-ΔC-His
84R1)
26.7 (1.6) Å
31.5 (7.3) Å
43.5 (10.6) Å
26.8 (2.1) Å
34.0 (12.7) Å
55.1 (20.2) Å
P (r)
time(μs)
distance (Å)
time(μs)
distance (Å)

Figure S3.

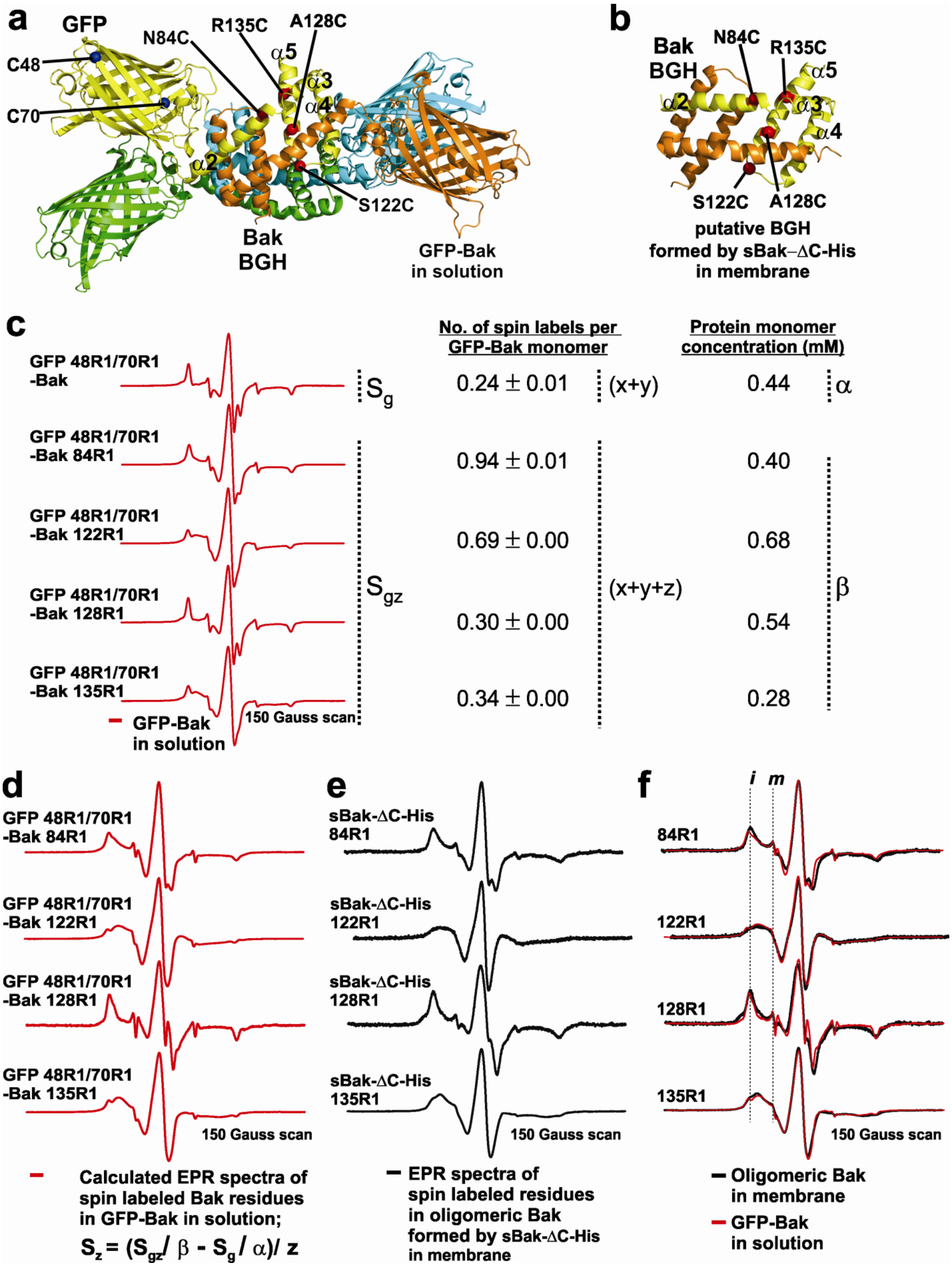
a
GFP
C48
C70
N84C
R135C
A128C
α5
α3
α4
α2
S122C
Bak
BGH
GFP-Bak
in solution
b
Bak
BGH
N84C
R135C
α5
α2
α3
α4
S122C
A128C
putative BGH
formed by sBak–ΔC-His
in membrane
c
No. of spin labels per GFP-Bak monomer
Protein monomer concentration (mM)
GFP 48R1/70R1 -Bak
Sg
0.24 ± 0.01
(x+y)
0.44
α
GFP 48R1/70R1 -Bak 84R1
0.94 ± 0.01
0.40
GFP 48R1/70R1 -Bak 122R1
0.69 ± 0.00
0.68
Sgz
(x+y+z)
β
GFP 48R1/70R1 -Bak 128R1
0.30 ± 0.00
0.54
GFP 48R1/70R1 -Bak 135R1
0.34 ± 0.00
0.28
GFP-Bak in solution
150 Gauss scan
d
GFP 48R1/70R1 -Bak 84R1
GFP 48R1/70R1 -Bak 122R1
GFP 48R1/70R1 -Bak 128R1
GFP 48R1/70R1 -Bak 135R1
150 Gauss scan
Calculated EPR spectra of spin labeled Bak residues in GFP-Bak in solution;
Sz = (Sgz/ β - Sg / α)/ z
e
sBak-ΔC-His 84R1
sBak-ΔC-His 122R1
sBak-ΔC-His 128R1
sBak-ΔC-His 135R1
150 Gauss scan
EPR spectra of spin labeled residues in oligomeric Bak formed by sBak-ΔC-His in membrane
f
i
m
84R1
122R1
128R1
135R1
150 Gauss scan
Oligomeric Bak in membrane
GFP-Bak in solution

Figure S4.

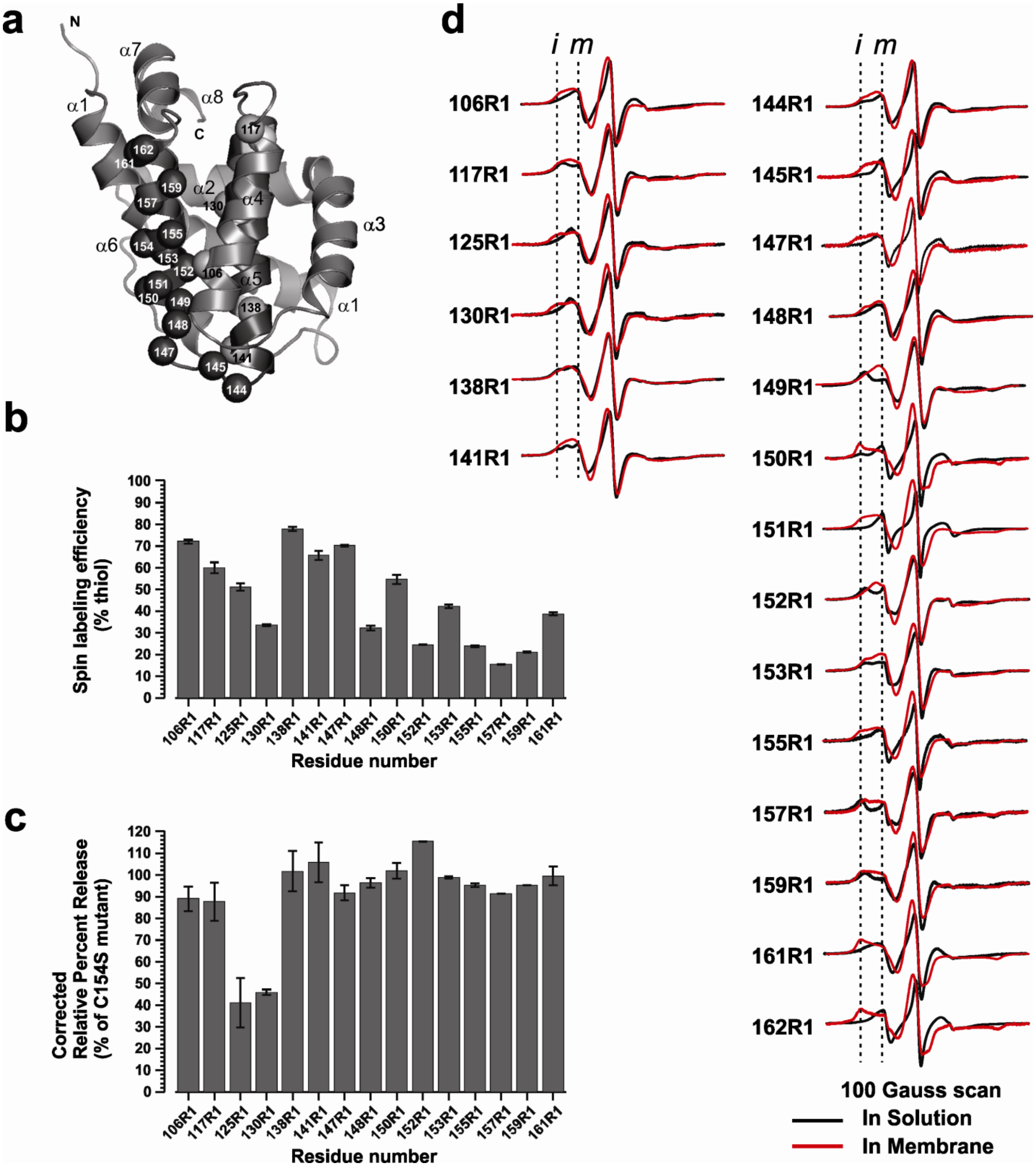
a
b
Spin labeling efficiency (% thiol)
Residue number
c
Corrected Relative Percent Release (% of C154S mutant)
Residue number
d
100 Gauss scan
In Solution
In Membrane

Figure S5.

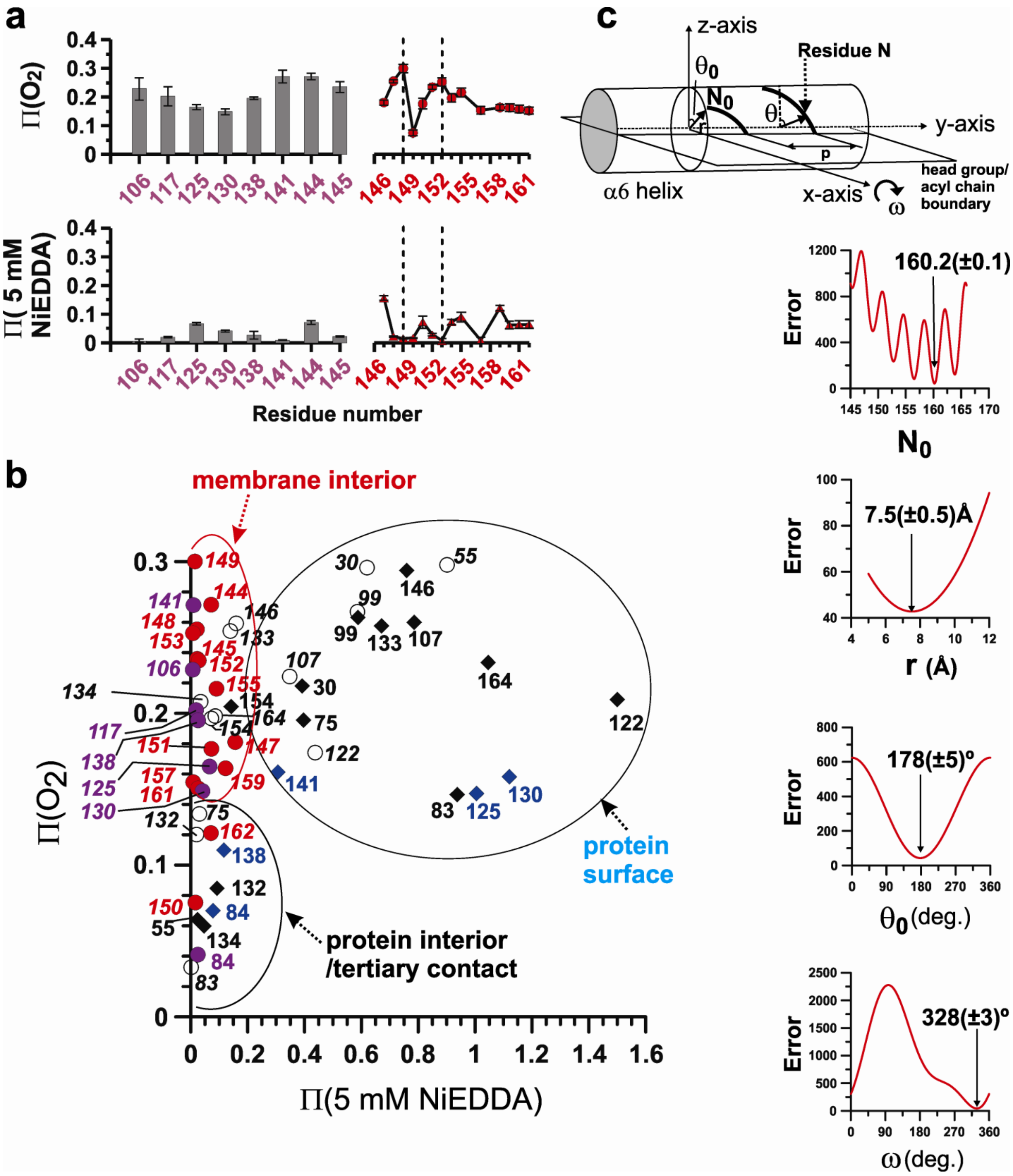

a
c
z-axis
Residue N
y-axis
x-axis
α6 helix
head group/
acyl chain
boundary
Residue number
160.2(±0.1)
7.5(±0.5)Å
178(±5)°
328(±3)°
Error
b
membrane interior
protein surface
protein interior
/tertiary contact
Π(5 mM NiEDDA)

Figure S6.

Supplemental Figure Legends

Figure S1. Amino acid sequences of Bax and Bak, crystal packing of GFP-Bak tetramers and Copper(II)(1,10-phenanthroline)$_3$-catalyzed cross-linking of various Bak cysteine mutants via disulfide bonds in mitochondrial apoptotic Bak oligomeric pore. (a) Amino acid sequence alignment of Bax and Bak for human and mouse. The sequences were aligned with Clustal Omega (http://www.ebi.ac.uk/Tools/msa/clustalo/). The sequences are shaded in yellow for the indicated α-helices (The sequences for Bax α3 helices were underlined). BH1-3 over the sequences represent the Bcl-2 homology domains 1-3. **(b) The crystal packing of the GFP-Bak tatramers is mediated only by interactions between the GFP molecules.** GFP-Bak tetramers crystallized into the $P3_121$ space group (See Table 1). Three symmetry-related GFP-Bak tetramers making contacts in the crystal are shown along the indicated 3-fold screw axis, to which the crystallographic unit cell (green diamond) viewed along the ***c*** axis (black dot) is superimposed. Only one of the two screw axes in the unit cell is shown for clarity. Note that only the GFPs are involved in the crystal contacts but not the Bak BGHs in each tetramer. **(c) Copper(II)(1,10-phenanthroline)$_3$-catalyzed disulfide bond formation in various Bak cysteine mutants expressed in mouse embryonic fibroblast mitochondria with or without activation by 100 nM p7/p15 Bid** (also see Figure 2f-h). The western blotting images of the samples cross-linked in the presence (+) or absence (-) of 100 nM p7/p15 Bid were obtained after SDS PAGE under a reducing (lower panel) or nonreducing (upper panel) condition (~30 μg mitochondrial proteins/lane). Monomer (M), dimer (D), and higher order oligomers (4x and 6x) are indicated along with the PAGE molecular weight standards. The asterisk symbol represents a band that reacted to the Bak antibody nonspecifically. The inset on the right-hand side in the upper panel represents lane 6 after adjustment of the tone to highlight the Bak bands.

Figure S2. Spin labeling efficiency, membrane permeabilizing activity, accessibility parameters and electron paramagnetic resonance (EPR) spectra of sBak-ΔC-His /84R1. (a) sBak-ΔC-His N84C mutant protein was labeled efficiently with the spin label and the spin labeled residue (84R1) did not interfere with the membrane-permeabilizing activity of Bak significantly. A C-terminally hexahistidine tagged soluble form of mouse Bak (residues 16-184, designated as sBak-ΔC-His)[1] was spin labeled at residue 84 (designated as sBak-ΔC-His/84R1**)** after introducing a cysteine substitution mutation as described (*ibid*). The efficiency of spin labeling for the indicated residues (percent of thiols labeled, left bar) was determined using 3-carboxyproxyl (Sigma-Aldrich) as a standard as described[2]. Corrected relative percent release activity (%) (right bar in the graph) was determined as described in Supplemental Methods. Average values of two experiments are shown with the error ranges indicated. **(b) Accessibility parameters of air oxygen, Π(O_2), and 5 mM NiEDDA, Π(5 mM NiEDDA), to 84R1 in Bak indicate that 84R1 is located in solvent-inaccessible protein interior in solution and in membrane-inserted states of Bak.** The accessibility parameters were determined in solution state (18% (v/v) glycerol) and in membrane-inserted Bak as described[1]. When the values were plotted in Π(O_2) vs. Π(5 mM NiEDDA) plot in Supplemental Figure S6b, the data point was positioned in 'protein interior/tertiary contact' **(**the blue diamond for solution state and the purple dots for membrane-inserted state labeled as 84). This indicated that 84R1 is located in the protein interior or at a tertiary contact site both in the solution state and in the membrane-inserted state (*i.e.*, in oligomeric Bak). Of note, residue N84 is located in protein interior in the homology model of soluble Bak[1] (see Fig 3B), lending credence to our data interpretation. **(c) EPR spectra of 84R1 indicate that the residue is immobile both in solution state and membrane-inserted state of Bak.** The X-band EPR spectra of 84R1 were recorded at room temperature in solution state and in membrane-inserted state of Bak. The spectra indicated that the nitroxide spin label is very immobile

both in solution and in membrane-inserted state of Bak (sBak-ΔC-His). The $2A_{zz}$ value increased by 2.6 Gauss in the membrane-inserted state in comparison to the solution state, indicating that the mobility of the spin label decreased further in oligomeric Bak. **(d) Relative positions of two pairs of spin labels can be triangulated using the inter-spin distances if they are assumed to be on the same plane.** For the two spin label pairs located at ***a***, ***b***, ***a'*** and ***b'*** as shown in Fig 3c, a convenient Cartesian coordinate can be chosen to describe their positions, where ***b*** on y-axis is at a distance d_1 from ***a*** that is at the origin and ***a'*** and ***b'*** are located as shown at the indicated distances from each other for two different arrangement of the two spin label pairs (Cases 1 and 2). Then the x, y coordinates of the positions in the Cartesian coordinate system can be written as shown. Using the distance relationship, the x and y (or x' and y') values can be calculated using the Pythagorean theorem. For case 1, $x = [d_3^2 - \{(d_3^2+d_1^2-d_2^2)/(2d_1)\}^2]^{1/2}$, and $y = (d_3^2+d_1^2-d_2^2)/(2d_1)$. For case 2, $x' = [d_2^2 - \{(d_1^2+d_2^2-d_3^2)/(2d_1)\}^2]^{1/2}$, and $y' = (d_1^2+d_2^2-d_3^2)/(2d_1)$. These two solutions are redundant to their counterparts obtained by the reflection operation relative to x- and/or y-axes. For the set of distances from Fig 3e (d_1 = 26.6 (5.2) Å, d_2 = 32.6 (3.6) Å, and d_3 = 49.4 (5.2) Å); x = 30.1 Å, y = 39.2 Å, x'=30.1 Å, and y'= -12.6 Å. With these, the coordinates of ***a***, ***a'***, ***b*** and ***b'*** can be calculated as shown (see Fig 3g). If the set of distances from Q-band data analyzed by DEFit program is used (d_1 = 26.8 (2.1) Å, d_2 = 34.0 (12.7) Å, and d_3 = 55.1 (20.2) Å; see Fig S3c), x = 26.2 Å, y = 48.5 Å, x'=26.2 Å, and y'= -21.7 Å, respectively. Modeling showed that only case 1 was consistent with the 'α3/α5 interface,' which is shown in Figure 3g.

Figure S3. The distance between two 84R1s, that are present in BGH domains in GFP-Bak, can be determined by double electron electron resonance (DEER) method. (a) Locations of R1 residues and their inter-spin interactions in GFP-Bak tetramer in solution. Among all the possible spin label pairs in a GFP-Bak tetramer, *e.g.*, those within GFPs (C48R1 and C70R1) or BGHs (84R1-84R1'), or between any of these domains, only the shortest inter-spin distance within the detection limit of DEER for each category is represented as the C_α-C_α distance. Note that the C_α-C_α distance of ~16 Å for 84R1s within the BGH domains is the shortest among all the possible pairs of the spin labels. **(b) Inter-spin distances in two neighboring BGHs within oligomeric Bak.** The C_α-atoms of 84R1s are shown in red spheres with the intra-dimer and the inter-dimer spin-spin interactions indicated by solid red and dashed red arrows, respectively. Of note, only the two closest inter-dimer spin-spin interactions are shown for clarity. **(c) The inter-spin distances, determined by DEFit program[3] for the DEER data for spin labeled GFP-Bak and oligomeric Bak samples, indicate that the distance between 84R1s within a BGH is ~27 Å.** DEER signals were obtained using X-band and Q-band DEER for the tetrameric spin-labeled wild-type GFP-Bak (designated as GFP 48R1/70R1-Bak), the tetrameric spin-labeled GFP-Bak 84C substitution mutant (designated as GFP 48R1/70R1-Bak/84R1), and oligomeric Bak prepared using sBak-ΔC-His/84R1. Background-subtracted X-band (first column) and Q-band (third column) DEER data are shown in red and blue solid traces, respectively. The superimposed black dotted lines represent theoretical fits obtained by analyzing the corresponding DEER data using the DEFit program[3] (See Fig 3). Their resulting distance distribution probability functions are shown in the second and fourth columns, respectively, with the mean value of the distance distribution and the width at half-height in parenthesis indicated for each corresponding peak. The reliability levels of the distance distributions are shaded in pale gray and dark gray[4], in which the latter would be unreliable if the DEER data were analyzed by DeerAnalysis2013 [5]. Of note, the total spin labels in GFP 48R1/70R1-Bak and GFP 48R1/70R1-Bak/84R1were 0.24 (±0.01) and 0.94 (±0.01), respectively (see Supplemental Figure S3c), which made the DEER modulation by 84R1s detectable over the GFP background signal. The data for oligomeric Bak (4th row) are identical to those shown in Fig 3d & e, which were analyzed here by DEFit program [3] for comparison with the results by DeerAnalysis 2013 program[5]. Analysis of the data by the Global Analysis

program[6] also gave similar results (not shown). **(d) The MMM 2010 program[7] could not identify a solvent-inaccessible and immobile rotamer of 84R1 in BGH although it predicted the range of inter-spin distances comparable to the experimental results**. *Top panel:* Using the MMM program[7], a library of rotamers of the 84R1 residue was generated at the two symmetry-related locations of 84R1 in the BGH structure (using A & B chains in Figure 1d, left panel). Majority of these rotamers of 84R1 generated by the MMM program were exposed to water phase, contrary to what the accessibility measurements indicated (see Supplemental Figure S2b). *Bottom panel:* The range of possible distances between them was also calculated using the same program, which predicted that the most probable inter-spin distance is ~27 Å as shown. **(e) Rotamers of 84R1 that are immobile and inaccessible to collision reagents were identified by manual modeling using Pymol[8], Coot[9] and MOE programs[10].** *Top panel:* The side chains of D88, Q92, R135, L138 and Y134 in polypeptide chain A were rearranged to create a pocket for the spin label 84R1 on chain A, designated as 84R1 (A), as shown. In addition, residues S122 and W123 on the other polypeptide chain (B) were rearranged. After spin label 84R1 was placed in the pocket, energy minimization was performed in MOE. The nitroxide methyl groups were within the range of van der Walls interactions with the hydrocarbon moieties from the surrounding residues. The NO group of the nitroxide spin label was well sequestered from the surface and had very limited space for tumbling motion, thus keeping it in an 'immobile' state (See Fig 3f). The symmetry-related spin label 84R1' on chain B, designated as 84R1 (B), and its surrounding residues were modeled identically. In 84R1 (A), the carbon atoms are colored in green, sulfur atoms are in yellow. In the surrounding side chains, the carbon atoms are in cyan, nitrogen atoms are in blue. The hydrogen and oxygen atoms are in white and in red, respectively, in the spin label and all the listed side chains. For clarity, other parts are shown in dark gray. *Bottom panel:* The nitrogen to nitrogen distance between 84R1 (A) and 84R1 (B) in BGH was 23.7 Å, which was 2.9 Å shorter compared to the measured distance of 26.6 Å (d_1) (Fig 3e). Spin labels are colored in the same way as in the upper panel (also see Figure 3f).

Figure S4. The X-band EPR spectra of spin labels attached to the BGH domains at selected locations remain essentially the same whether the domains are present in the tetrameric GFP-Bak in solution or in oligomeric Bak in membrane. (a) Residues N84, S122, A128 and R135 located in the BGH domains in the tetrameric GFP-Bak in solution were selected for site-directed spin labeling. The four locations in Bak BGH domain selected for site-directed spin labeling (SDSL) are as follows; N84 on the α3 helical surface, S122 in an exposed part of α4-5 loop, A128 on the α5 helix in protein interior, and R135 on the α5 helix at an exposed tertiary contact site in the tetrameric GFP-Bak model. For clarity, the C_α atoms of R1s are shown in blue and red spheres in GFP and Bak (mouse Bak α2-α5 helices, residues 66-144), respectively, in only one polypeptide chain (yellow). **(b) The putative locations of residues N84, S122, A128 and R135 in the BGH in oligomeric Bak, which were selected for site-directed spin labeling in sBak-ΔC-His.** The C_α atoms of the spin labeled residues in oligomeric Bak, corresponding to those indicated in *a*, are shown in red spheres in only one polypeptide chain of a BGH model. **(c) X-band EPR spectra of the tetrameric GFP-Bak spin labeled at 84C, 122C, 128C and 135C in BGH domains obtained in solution state, which contain the spectral components from 48R1 and 70R1 in GFP.** *Left panel:* EPR spectra were measured for the tetrameric GFP-Bak spin labeled at the indicated sites; residues 84C, 122C, 128C and 135C in mouse Bak α2-α5 domain and/or residues 48C and 70C in GFP. S_g and S_{gz} represent the spectra of the indicated samples before normalization, *i.e.*, raw data, all of which were recorded under the same experimental condition at room temperature. Shown here are the spectra normalized to the same peak-to-peak height for the central line for ease of comparison. *Middle panel:* The total number of spin labels attached to each indicated GFP-Bak monomer was determined as described in the Supplemental Methods (averages of two measurements). We assumed

that an equal spin labeling efficiency (x, y, and z) was achieved for the identical locations in different mutant proteins under the spin labeling condition described in the Supplemental Methods. *Right panel:* Protein concentrations were determined using a Nanodrop (Thermo Scientific) assuming that the average extinction coefficient of the fusion proteins is 33,350 $M^{-1}cm^{-1}$. The typical error range was ±1%. The symbols α and β represent the monomer concentration of the indicated proteins. **(d) X-band EPR spectra of the tetrameric GFP-Bak spin labeled at 84C, 122C, 128C and 135C in BGH domains obtained by spectral subtraction.** Due to lack of a cysteine-less GFP construct, we attempted to obtain the EPR spectra of the spin labels attached to BGH domains in GFP-Bak by spectral subtraction. S_z, the calculated spectrum for an individual spin label attached to BGHs, is given by $[S_{gz}/\beta\text{-}S_g/\alpha]/z$. Shown here are the S_z spectra after normalization to the same peak-to-peak height for the central line. **(e) X-band EPR spectra of 84R1, 122R1, 128R1 and 135R1 in the oligomeric Bak in membrane.** The oligomeric Bak samples were prepared using sBak-ΔC-His samples individually spin labeled at 84C, 122C, 128C or 135C as described in the Supplemental Methods. With these, the corresponding oligomeric Bak samples were made and the EPR spectra were recorded. Spectra shown were normalized to the same peak-to-peak height for the central line. Of note, the spectra for 84R1 shown here and in Supplemental Figure S2c were obtained from two separate experiments. **(f) The X-band EPR spectra of the four R1 residues in BGH for the tetrameric GFP-Bak in solution were superimposable to their corresponding spectra for oligomeric Bak in membrane, indicating that the local molecular environment around each R1 residue remained almost the same whether the BGH existed in the tetramertic GFP-Bak in solution or in oligomeric Bak in membrane.** The spectra shown in *d* and *e* were superimposed for the indicated R1s. The 'immobile' and 'mobile' components of the spectra are denoted by 'i' and 'm' on top of the dotted vertical lines in the corresponding regions. Consistent with its location in the BGH model, 128R1 displayed 'immobile' feature in the spectra. The surface residue 84R1 also displayed pronounced 'immobile' features in the spectra despite its apparent location on the surface of helix α3. The results indicated that 84R1 side chain was located in the 'protein interior' location merely by the folding of Bak α2-α5 domains in BGH even in the absence of helices α6-α8 (GFP-Bak contains helices α2-α5 only).

Figure S5. Site directed spin labeling in Bak helices α4-α6. (a) Sites of site-directed spin labeling in sBak-ΔC-His. In a homology model of sBak-ΔC-His[1], the amino acid residue locations are indicated on the spheres representing the C_α-atoms selected for single cysteine substitution mutation and spin labeling. **(b) Spin labeling efficiency of cysteine residues introduced in sBak-ΔC-His.** The mutant sBak-ΔC-His proteins individually spin labeled with the MTSSL spin label (Figure 3a) were prepared as described in the Supplemental Methods. The efficiency of spin labeling for the indicated residues are the average values of two experiments with the error ranges indicated. **(c) Corrected relative percent release activity of spin labeled sBak-ΔC-His.** The average values and the indicated error ranges of the corrected relative percent release activity (%) were determined by two duplicate liposome dye release assays (See Supplemental Methods). **(d)** X-band **EPR spectra of spin labeled sBak-ΔC-His in solution state (inactive monomeric Bak) and in membrane-inserted state (oligomeric Bak).** The EPR spectra of the Bak samples in solution state (thick black traces) and in membrane-inserted state (thin red traces) were normalized to the same central peak height. All the spectra were obtained at room temperature by multiple scans over the 100 Gauss window. The two vertical dotted lines represent the EPR spectral features representing mobile ('*m*') or immobile ('*i*') component of the spin label population. *Note:* In *b* and *c*, the values for 144R1, 145R1, 149R1, 151R1, 154R1, and 162R1 are not shown since they were reported earlier[11]. In *d*, the membrane spectra for 144R1 and 145R1 were reported earlier (*ibid*), but are shown here for comparison with the solution spectra. The immersion depths of the R1s were selectively

measured among the oligomeric Bak samples of which the EPR spectra display ‘mobile’ or relatively ‘mobile’ features (See Figures 4 and S6).

Figure S6. Accessibility parameters of spin labeled residues in membrane-inserted sBak-ΔC-His (oligomeric Bak) and their topological locations. (a) Accessibility parameters of air oxygen, $\Pi(O_2)$, and 5 mM NiEDDA, Π(5 mM NiEDDA), to the spin-labeled residues. The accessibility parameters of the spin labeled residues in the membrane-inserted sBak-ΔC-His are shown as a function of residue locations. The membrane-inserted sBak-ΔC-His samples were prepared as described in Supplemental Methods. For residues 146-162 in helix α6 (residues 149-163), the locations that display local maxima of $\Pi(O_2)$, and their corresponding positions in Π(5 mM NiEDDA) vs. residue plot are marked with vertical dotted lines. Average values of 2-3 experiments are shown with the error ranges indicated. **(b) Mapping the topological locations of spin labeled residues from a two-dimensional plot of accessibility parameters $\Pi(O_2)$ and Π(NiEDDA)**. To map the topological locations of the R1 residues listed in *a*, their accessibility parameters were superimposed in a 2-dimensional plot to those of known topological information[1]. Briefly, open circles and black diamonds represent the accessibility parameters of air oxygen and NiEDDA (5 mM) for the indicated R1s in sBak-ΔC-His protein in membrane-inserted state and in solution, respectively, which were categorized into three groups; ‘*protein interior/tertiary contact*’, ‘*protein surface*’, or ‘*membrane interior*’ with their corresponding boundaries in the 2-d plot approximately defined as shown[1]. Using this as a reference, the residues shown in *a*, represented by the circles filled with purple (residues 106-145) or red color (residues 146-162) in the 2-d plot, were categorized into two groups clearly; ‘membrane interior’ or ‘protein interior/tertiary contact.’ The blue diamonds represent the data for solution state for certain selected residues. Of note, residue 147, located in ‘membrane interior’ region near the boundary of the ‘protein surface’ region, had a much bigger accessibility value than other ‘membrane interior’ residues when 50 mM NiEDDA was used (data not shown). This indicated that residue 147 was in fact exposed to water (see Figures 3c and 3d). The residues identified to be in the ‘membrane interior’ were further analyzed for immersion depth determination with 50 mM NiEDDA as described in Material and Methods. The results are summarized in Figure 3a. The accessibility data for 84R1 from Supplemental Figure S2b are also plotted here (blue diamond for solution state and the purple dot for the membrane-inserted state). **(c) Fitting of the immersion depths of R1 residues in helix α6.** The experimental depths of α6 residues in Figure 4a were fitted to a theoretical curve by minimizing the error term as described in the Supplemental Methods. Since residue 157R1 displayed a lineshape with an immobile component (Supplemental Figure S5d), possibly due to tertiary interactions that could skew the depth measurement, the data fitting was attempted with (solid black curve in Figure 4a) or without it (red dotted curve in Figure 4a). Both gave similar results (see Figure 4a). The best fitting parameters were; $\boldsymbol{N_0}$ = 160.2 (±0.1), $\boldsymbol{r}$ = 8.0 (±0.5) Å, $\boldsymbol{\theta_0}$ = 175 (±5)°, and $\boldsymbol{\omega}$ = 327 (±3)° when residue 157 was included (The Error functions are not shown here) (see the black solid curve in Figure 3a). Excluding it, the parameters were; $\boldsymbol{N_0}$ = 160.2 (±0.1), $\boldsymbol{r}$ = 7.5 (±0.5) Å, $\boldsymbol{\theta_0}$ = 178 (±5)°, and $\boldsymbol{\omega}$ = 328 (±3)° (red dotted curve in Figure 3a; also see the Error functions shown here). The error ranges of the parameters were defined as the range of parameters that gave the error value 2% larger than the converged minimum error. For the latter fit, the value $\boldsymbol{\omega}$ of 328 (±3)° means that the helix is rotated around the x-axis clockwise by 328 (±3)°, equivalent to a counterclockwise rotation by 32 (±3)°, indicating that the helix is tilted toward the N-terminus by ~30° as shown in Figure 4d. The parameters $\boldsymbol{N_0}$ = 160.2 (±0.1) and $\boldsymbol{\theta_0}$ = 178 (±5)° define the rotational orientation of the helix around the helical axis. Since $(\boldsymbol{\theta} - \boldsymbol{\theta_0}) = 100\ (\boldsymbol{N} - \boldsymbol{N_0})$ in an α-helix, $\boldsymbol{\theta} = \boldsymbol{\theta_0} + 100\ (\boldsymbol{N}-\boldsymbol{N_0})$. Thus, for $\boldsymbol{N}$=160 and $\boldsymbol{\theta_0}$ = 178°, $\boldsymbol{\theta}$ = 178 (±5)° + 100(160-160.2) = 158 (±5)° = (180 - 22) (±5)°. Thus, the radial vector of residue 160 is -22

(±5)° away from the direction of the greatest depth (Figure 4c). Note that the angle between two neighboring radial vectors on a helical diagram is 20° (= 360/18). This is the reason why the direction of the greatest depth was drawn on the left of residue 153. Considering the experimental errors in $\boldsymbol{\theta_0}$ value, the direction of the greatest depth will pass between residues 146 and 153 in the helical wheel diagram (Figure 4c). Additionally, the parameter $\boldsymbol{N_0}$ = 160.2 (±0.1) means that the helical axis at this position intercepts the hypothetical plane between the lipid head group and the hydrocarbon chain region, indicating that the helical axis at residue 162 is located just below the acyl chain/head group interface (This was not depicted in Figure 4d). Of note, the values plotted in Figure 4a represent the depths from the surface of the membrane, *i.e.*, Depth ($\boldsymbol{N}$, $\boldsymbol{N_0}$, $\boldsymbol{r}$, $\boldsymbol{\theta_0}$, $\boldsymbol{\omega}$, $\boldsymbol{p}$) + 5 Å, assuming that the thickness of the head group is ~5 Å. This figure was adapted from Oh et al.[12].

Supplemental Methods

Crystallization of GFP-Bak and data collection. Purified GFP-Bak was crystallized by hanging-drop vapor diffusion method: 1 μl of 20 mg/ml protein was mixed with 1 μl of precipitating solution (7-12% PEG3350, 20% MPD, 100 mM Tris (pH 7.0-8.5), 0.5% CHAPS). Rod-like crystals of 50 μm · 50 μm · 100 μm size appeared within 7 days at 8% (w/v) PEG3350, 20% (v/v) MPD, 100 mM Tris (pH 7.5), and 0.5% (w/v) CHAPS at 18°C. To improve crystal diffraction protein, crystals were dehydrated in a precipitant concentration of 30% (v/v) MPD, 12% (w/v) PEG3350. Crystals were flash-frozen in liquid nitrogen and diffraction data were collected at 100 K at the GM/CA-CAT, Advanced Photon Sources in Argonne National Laboratory.

X-ray crystal structure determination. The X-ray diffraction data were processed with HKL2000[13]. The structure was solved by molecular replacement (MR) with PHASER[14], using the GFP part of PDB ID 4BDU[15] as a search model. The MR solution revealed electron density for the helical regions of Bak. Then, the Bax BGH structure of PDB ID 4BDU was used as a search model in PHASER. Model building for the mouse Bak structure was done in COOT[16] based on the electron density determined in the two rounds of MR. Successive rounds of model building and structure refinement with PHENIX[17] and REFMAC[18] resulted in the structure of the tetrameric GFP-Bak presented in Table 1.

Disulfide cross-linking experiment. Copper(II)(1,10-phenanthroline)$_3$ (CuPhe)-mediated disulfide cross-linking experiments were carried out with the mitochondrial samples (containing 60 μg mitochondrial proteins) as described in the Methods section[19]**.** Mitochondria prepared in the trehalose buffer (300 mM trehalose, 10 mM KCl, 1 mM EGTA, 10 mM HEPES, pH 7.4) were spun down at 12,000 g for 10 min at 4°C. They were then resuspended in 100 μl of the cytochrome *c* release assay buffer (20 mM HEPES/KOH pH 7.5, 100 mM sucrose, 80 mM KCl, 1 mM ATP, 80 μM ADP, 5 mM Na Succinate, 1 mM DL-dithiothreitol (DTT)) in the presence of 0 or 100 nM p7/p15 Bid, and further incubated for 30 min at 30 °C. The samples were centrifuged at 12,000 g for 10 min at 4°C. The resulting pellets were resuspended in a volume of 30 μl cross-linking buffer (20 mM HEPES/KOH pH 7.5, 150 mM KCl, 100 mM sucrose, 5 mM $MgCl_2$, 2 mM $NaAsO_2$, 1.5 mM copper sulfate, 5 mM 1,10-phenanthroline) and further incubated for 30 min on ice. The reaction was quenched by mixing the reaction mixture with an equal volume of 2x nonreducing SDS-sample buffer containing 10 mM EDTA (125 mM Tris-HCl pH 6.8, 4 % (w/v) sodium dodecylsulfate (SDS), 25% (v/v) glycerol, 0.1% (w/v) bromophenol blue, 10 mM EDTA). For reducing gel electrophoresis, samples (10 μl) were mixed with 1/5th volume (2 μl) of 6x SDS buffer (375 mM Tris-HCl pH 6.8, 12% (w/v) SDS, 60% (v/v) glycerol, 0.6 M DTT, 0.06% (w/v) bromophenol blue). Samples (a quantity of 10 μg mitochondrial protein) were analyzed by SDS-PAGE, followed by immunoblotting. The primary and the secondary antibodies used were rabbit polyclonal anti-BAK aa23–38 antibody (Millipore, Cat. # 06-536) and HRP-conjugated goat anti-mouse antibody (Santa Cruz, Cat. # sc-2062).

Expression, purification and spin labeling of GFP-Bak cysteine substitution mutant proteins. Cysteine substitution mutations were introduced into the mouse Bak gene sequence (coding for α-helices 2-5, residues 66-144) by site-directed mutagenesis with the KOD Hot Start DNA Polymerase kit (cat no. 71086-3. Millipore) using the pYEGFP_A206N _BAK_H2-H5_pET28a plasmid (see Methods section) as a template. Here, the wild-type GFP (green fluorescent protein, residues 1-230) has two naturally occurring cysteines at residue locations 48 and 70 (designated as GFP 48C/70C), which could not be substituted to other amino acid residues without destabilizing the protein. The wild-type mouse Bak sequence (residues 66-144) does not have any naturally occurring cysteine. The single cysteine

substitution mutations introduced into this sequence were 84C, 122C, 128C and 135C by the mouse Bak amino acid sequence designation. The resulting GFP-Bak proteins were designated as GFP 48C/70C-Bak 84C, etc. Wild type or cysteine mutant GFP-Bak proteins were expressed as an N-terminally hexahistidine tagged fusion protein (designated as His-GFP-Bak) and purified by the same method as described in Methods. To achieve the maximum possible spin labeling efficiency for each cysteine, His-GFP-Bak proteins were spin labeled as follows; 20-fold molar excess of spin label was used relative to the same concentration of cysteines in reaction mixture for 15 hrs at room temperature, specifically, 1.08 mM MTSSL spin label in the presence of 0.054 mM protein cysteines (*i.e.*, 1.0 mg/ml for wild-type His-GFP-Bak and 0.67 mg/ml for cysteine mutant proteins in 20 mM Tris, 150 mM NaCl, pH 8 (**TBS**)). The unreacted spin label was removed by gel filtration using the Superdex 200 10/300 column (GE Healthcare) with TBS as an eluant at a flow rate of 0.5 ml/min at room temperature. Spin-labeled proteins were further concentrated using centrifugal concentrators (molecular mass cutoff of 50 kDa, Millipore). The protein concentrations were determined by measuring the absorbance at 280 nm using a Nanodrop (ThermoScientific) assuming that the average molar extinction coefficient of the proteins is 33,350 $M^{-1}cm^{-1}$. The efficiency of spin labeling for the indicated residues was determined using 3-carboxyproxyl (Sigma-Aldrich) as a standard as described[2]. Briefly, the spin labels were liberated from the protein by treating the protein solution with an equal volume of 100mM tris(2-carboxyethyl)phosphine for 30min at room temperature. The central peak heights of the EPR spectra were measured for the samples and the 3-carboxyproxyl standards (10-100 μM) in the same volume of 10 μls. The concentrations of spin labels in the samples were determined from the standard curve.

Preparation of sBak-ΔC-His cysteine substitution mutant proteins and spin labeling. Cysteine substitution mutant genes of a soluble form of mouse Bak (residues 16-184) was prepared by site-directed mutagenesis using the QuickChange site-directed mutagenesis kit (Stratagene) using the pPosKJ-sBAKC154S-CHis plasmid[1]. Using the mutant plasmids, the mouse Bak mutant proteins were expressed in the cytoplasm of *E. coli* BL21(DE3) as a fusion protein with an N-terminal bacterial hemoglobin and a C-terminal hexahistidine tag (Hb-sBak-ΔC-His) as described[1]. The hemoglobin tag was cleaved by digestion with glutathione S-transferase tagged tobacco etch virus protease (GST-TEV 219V) and the sBak-ΔC-His proteins were purified by using a semi-automated two step Ni^{2+}-NTA-affinity/anion exchange chromatography as described[1]. A few milligrams of single cysteine mutants of sBak-ΔC-His were spin labeled with 10-20-fold molar excess (0.25-1.5 mM) of (1-oxyl-2,2,5,5,-tetramethyl-Δ3-pyroline-3-methyl) methanethiosulfonate spin label (MTSSL) (Toronto Research Chemicals, Inc., Toronto, Canada) in buffer A at room temperature overnight (~16 h). Unreacted MTSSL was removed by gel filtration using a Superdex 75 10/30 (GE Healthcare) in buffer A at a flow rate of 0.5 ml/min. If needed, spin-labeled proteins were further concentrated using centrifugal concentrators with a molecular cutoff of 10 kDa (Millipore) at 4°C. The efficiency of spin labeling for the indicated residues were determined using 3-carboxyproxyl (Sigma-Aldrich) as a standard as described above. Glycerol was added to the proteins samples to a final concentration of 18% (v/v) for storage at -80°C.

Preparation of Liposome preparation. Large unilamellar vesicles (LUVs) mimicking the lipid composition of the mitochondrial outer membrane contact sites[20,21], were made with a mixture of 1-palmitoyl-2-oleoyl-*sn*-glycero-3-phosphocholine (POPC), 1-palmitoyl-2-oleoyl-*sn*-glycero-3-phosphoethanolamine (POPE), beef liver phosphatidylinositol (PI), beef heart cardiolipin, cholesterol and 1,2-dioleoyl-*sn*-glycero-3-{[*N*-5-amino-1-carboxylpentyl]iminodiacetic acid}succinyl} (nickel salt) (DOGS-NTA-Ni) (all from Avanti Polar Lipids, Inc.) at a weight ratio of 36: 22: 9: 8: 20: 5 in 20 mM HEPES, 150 mM KCl (pH 7.0) (**buffer A**) as described[2]. DOGS-NTA-Ni, a metal chelator lipid analog,

was included to bind the His-tagged soluble Bak or His-tagged p7/p15 Bid to the membrane surface efficiently[1,2]. The mixture of lipids in chloroform was prepared in clean borosilicate glass test tubes (13 x 100 mm, Fisherbrand) in 25 or 50 mg aliquots and the lipids were dried as a thin film by evaporating chloroform under a nitrogen gas flow using a glass pasteur pipet in the hood. The film was further dried overnight in a desiccators connected to a vacuum pump in a hood. The dried lipids in test tubes were individually sealed in plastic pouches filled with nitrogen gas and kept at -80°C. To make unilamellar vesicles, the lipids were warmed to room temperature and emulsified in buffer A by vortexing. The emulsion was then freeze-thawed using liquid nitrogen and room temperature water to break the large multilamellar vesicles (MLVs) into smaller sized ones. The resulting MLVs were then extruded 15 times through two sheets of polycarbonate membranes with a pore diameter of 100nm (Avanti Polar Lipids), resulting in translucent LUV solution. The LUVs encapsulating fluorescein isothiocyanate-dextran 10 (FITC-dextran, 10 kDa, Invitrogen) were also prepared with the same lipid composition[12,22-24]. Briefly, 50 mgs of FITC-dextran 10 was solubilized in 0.5 ml buffer A. This solution was added to the lipid film (25 mg) in a test tube and vortexed until the lipids were emulsified. The resulting MLVs were extruded as described above. The resulting LUVs were separated from free FITC-dextran by gel filtration using a 30 cm x 10 mm column prepared with Sephacryl 300 High Resolution gel (Cat no. 17-0599-01, GE Healthcare) in buffer A at 0.5 ml/min flow rate. The purified LUVs were stored in the presence of 18% (v/v) glycerol in 50 ml aliquots at -80°C as described. The liposomes were quantified by determining the phosphate content as described[25].

Liposomal dye release Assay. The extent of FITC-dextran 10 release from liposomes by spin labeled sBak-ΔC-His proteins (5 nM) were measured in presence of 25 nM N-terminally His-tagged p7/p15 Bid after a 25-min incubation at 37°C as described[2]. The release of the FITC-dextran 10 from the LUVs was monitored by fluorometry using a SLM 8000C fluorometer (Olis Instruments, Inc) in a thermostatted 1-cm path length quartz cuvette with constant stirring at 37 °C as described[2]. The extent of marker release was quantified on a percentage basis and the relative percent release of samples was then standardized against the reference as described[2]. Briefly, corrected relative percent release activity (%) (right bar in the graph) was approximated as follows as reported earlier[11]; Corrected relative percent release activity (%) = $\{T-(1-\chi)C\}/\chi$ where T is the relative percent release of the spin labeled sample, C is the relative percent release of the unlabeled protein, and χ is the fraction of the spin labeled protein in the sample (= spin labeling efficiency(%) / 100). In all assays, the concentration of LUVs was 10 μg/ml of lipids (or 0.125 nM). The relative percent release of each of the spin labeled proteins were further corrected by subtracting the contribution of the unlabeled cysteine substitution Bak protein present in the spin labeled samples as described[11].

Preparation of oligomeric Bak in membrane. The oligomeric Bak was prepared by modifying the procedures for the above liposome release assay as described[1]. Briefly, a total of 1 mg of LUVs prepared above in a total reaction volume of 150 μl was used to form oligomeric Bak pores with a total of 3.5 nmol of sBak-ΔC-His in the presence of 3.5 nmol p7/p15 Bid. For power saturation experiments, a mixture of spin labeled sBak-ΔC-His and unlabeled protein (sBak C154S-ΔC-His) at 3:4 ratio (designated as 3:4 mixture) was used to suppress the spin-spin interactions (See Figures S2b and 2c, S4e, S5d and S6a). For distance measurements by DEER, only the spin labeled protein (designated as 7:0 mixture) was used (See Figures 3d and 3e and S3c (4th row)). The following is the details of oligomeric Bak preparation described in Oh et al. [1]. First, sBak-ΔC-His solution (either a mixture of spin labeled protein and unlabeled protein (3:4 mixture) or purely spin labeled protein (7:0)) was added to the LUVs dropwise while vortexing it. After this, an equimolar (3.5 nmol) p7/p15 Bid was immediately added to the mixture and was incubated

for 45 min at 37°C. EDTA (0.5 M, pH 8) was added to the reaction mixture to a final concentration of 20 mM to stop binding of His-tagged proteins to the membrane by chelating the Ni^{2+} ions from the DOGS-NTA-Ni molecules incorporated into the liposomes. The LUVs were then spun down by centrifugation at ~110,000 x g for 15-20 min using an airfuge (Beckman). The liposome pellets were resuspended in 100 μl buffer A containing 5 mM EDTA and centrifuged one more time. EDTA was removed by washing the liposomes with 100 μl buffer A by two more cycles of resuspension and centrifugation. The resulting pellets were resuspended in a few microliters of buffer A for EPR experiments.

Fitting of the immersion depths. The following equation was used to describe the immersion depth of a spin label side chain R1 in an α-helix in a lipid bilayer as reported[12];

$$\text{Depth}(\boldsymbol{N}, \boldsymbol{N_0}, \boldsymbol{r}, \boldsymbol{\theta_0}, \boldsymbol{\omega}, \boldsymbol{p}) = \{(\boldsymbol{N}-\boldsymbol{N_0})\boldsymbol{p}/3.6\}\sin(\pi\boldsymbol{\omega}/180) - \boldsymbol{r}\cos(\pi\boldsymbol{\omega}/180)\cos\{5\pi(\boldsymbol{N}-\boldsymbol{N_0})/9 + \pi\boldsymbol{\theta}/180\},$$

where $\boldsymbol{N}$ represents the amino acid residue number; $\boldsymbol{N_0}$ represents the residue at which the helical axis intercepts the head group/hydrocarbon boundary plane (a hypothetical plane between the lipid head group and the lipid acyl chains); $\boldsymbol{r}$ represents the length of the nitroxide arm; $\boldsymbol{\theta_0}$ represents the rotational orientation angle in degrees of the $\boldsymbol{r}$ vector for residue $\boldsymbol{N_0}$ with respect to the membrane normal; $\boldsymbol{\omega}$ represents the helix tilting angle in degrees, and $\boldsymbol{p}$ represents the pitch of an α-helix, 5.41 Å per turn (of 3.6 residues). The best-fitting values of $\boldsymbol{N_0}$, $\boldsymbol{r}$, $\boldsymbol{\theta_0}$, and $\boldsymbol{\omega}$ were obtained by minimizing the error term, which is the sum of $[\text{Experimental depth}(\boldsymbol{N}) - \text{Depth}(\boldsymbol{N}, \boldsymbol{N_0}, \boldsymbol{r}, \boldsymbol{\theta_0}, \boldsymbol{\omega}, \boldsymbol{p})]^2$ for all $\boldsymbol{N}$s. Here, the Experimental depth ($\boldsymbol{N}$) was the measured depth of residue $\boldsymbol{N}$ relative to the head group/hydrocarbon boundary plane. The R program (version R 2.12.0)[26] was used to fit the data as described[12].

Supplemental References

1. Oh, K.J. et al. Conformational changes in BAK, a pore-forming proapoptotic Bcl-2 family member, upon membrane insertion and direct evidence for the existence of BH3-BH3 contact interface in BAK homo-oligomers. *J Biol Chem* **285**, 28924-37 (2010).
2. Oh, K.J. et al. A membrane-targeted BID BCL-2 homology 3 peptide is sufficient for high potency activation of BAX in vitro. *J Biol Chem* **281**, 36999-7008 (2006).
3. Sen, K.I., Logan, T.M. & Fajer, P.G. Protein dynamics and monomer-monomer interactions in AntR activation by electron paramagnetic resonance and double electron-electron resonance. *Biochemistry* **46**, 11639-49 (2007).
4. Jeschke, G. Interpretation of Dipolar EPR Data in Terms of Protein Structure. in *Structure and Bonding*, Vol. 152 83-120 (Springer-Verlag Berlin Heidelberg, 2011).
5. Jeschke, G. et al. DeerAnalysis2006 - a comprehensive software package for analyzing pulsed ELDOR data. *Appl. Magn. Res.* **30**, 473-498 (2006).
6. Brandon, S., Beth, A.H. & Hustedt, E.J. The global analysis of DEER data. *J Magn Reson* **218**, 93-104 (2012).
7. Polyhach, Y., Bordignon, E. & Jeschke, G. Rotamer libraries of spin labelled cysteines for protein studies. *Phys Chem Chem Phys* **13**, 2356-66 (2010).
8. DeLano, W.L. The PyMOL Molecular Graphics System. (DeLano Scientific, San Carlos, CA, USA, 2002).
9. Emsley, P. & Cowtan, K. Coot: model-building tools for molecular graphics. *Acta Crystallography* **D60**, 2126-2132 (2004).
10. Molecular Operating Environment (MOE), C.C.G.I., 1010 Sherbooke St. West, Suite #910, Montreal, QC, Canada, H3A 2R7, 2016.
11. Aluvila, S. et al. Organization of the mitochondrial apoptotic BAK pore: oligomerization of the BAK homodimers. *J Biol Chem* **289**, 2537-51 (2014).
12. Oh, K.J. et al. Conformational changes in BID, a pro-apoptotic BCL-2 family member, upon membrane binding. A site-directed spin labeling study. *J Biol Chem* **280**, 753-67 (2005).
13. Otwinowski, Z. & Minor, W. [20] Processing of X-ray diffraction data collected in oscillation mode. *Methods in Enzymology (Academic Press)* **276**, 307-326 (1997).
14. McCoy, A.J. et al. Phaser crystallographic software. *Journal of Applied Crystallography* **40**, 658-674 (2007).
15. Czabotar, P.E. et al. Bax crystal structures reveal how BH3 domains activate Bax and nucleate its oligomerization to induce apoptosis. *Cell* **152**, 519-31 (2013).
16. Emsley, P., Lohkamp, B., Scott, W.G. & Cowtan, K. Features and development of Coot. *Acta Crystallographica Section D Biological Crystallography* **66**, 486-501 (2010).
17. Adams, P.D. et al. PHENIX: a comprehensive Python-based system for macromolecular structure solution. *Acta Crystallographica* **D66**, 213-221 (2010).
18. Murshudov, G.N. et al. REFMAC5 for the refinement of macromolecular crystal structures. *Acta Crystallographica Section D: Biological Crystallography* **67**, 355-367 (2011).
19. Careaga, C.L. & Falke, J.J. Thermal motions of surface alpha-helices in the D-galactose chemosensory receptor. Detection by disulfide trapping. *J Mol Biol* **226**, 1219-35 (1992).
20. Lutter, M. et al. Cardiolipin provides specificity for targeting of tBid to mitochondria. *Nat Cell Biol* **2**, 754-61. (2000).
21. Ardail, D. et al. Mitochondrial contact sites. Lipid composition and dynamics. *J Biol Chem* **265**, 18797-802 (1990).
22. Terrones, O. et al. Lipidic pore formation by the concerted action of proapoptotic BAX and tBID. *J Biol Chem* **279**, 30081-91 (2004).
23. Kuwana, T. et al. BH3 domains of BH3-only proteins differentially regulate Bax-mediated mitochondrial membrane permeabilization both directly and indirectly. *Mol Cell* **17**, 525-35 (2005).
24. Kuwana, T. et al. Bid, Bax, and lipids cooperate to form supramolecular openings in the outer mitochondrial membrane. *Cell* **111**, 331-42 (2002).

25. Böttcher, C.J.F., gent, C.M.V. & Pries, C. A rapid and sensitive sub-micro phosphorus determination. *Analytica Chimica Acta* **24**, 203-204 (1961).
26. R Development Core Team. R: A language and environtment for statistical computing. Vienna, Austria: R Foundation for Statistical Computing. Retrieved from http://www/R-project.org. (2010).
